# Dynamic Metabolic Changes During Prolonged *Ex Situ* Heart Perfusion Are Associated With Myocardial Functional Decline

**DOI:** 10.3389/fimmu.2022.859506

**Published:** 2022-06-24

**Authors:** Mariola Olkowicz, Roberto Vanin Pinto Ribeiro, Frank Yu, Juglans Souto Alvarez, Liming Xin, Miao Yu, Roizar Rosales, Mitchell Brady Adamson, Ved Bissoondath, Ryszard T. Smolenski, Filio Billia, Mitesh Vallabh Badiwala, Janusz Pawliszyn

**Affiliations:** ^1^Department of Chemistry, University of Waterloo, Waterloo, ON, Canada; ^2^Jagiellonian Centre for Experimental Therapeutics (JCET), Jagiellonian University, Krakow, Poland; ^3^Division of Cardiovascular Surgery, Peter Munk Cardiac Center, Toronto General Hospital, University Health Network, Toronto, ON, Canada; ^4^Division of Cardiac Surgery, Department of Surgery, Faculty of Medicine, University of Toronto, Toronto, ON, Canada; ^5^Division of Cardiac Surgery, Department of Surgery, Dalhousie University, Halifax, NS, Canada; ^6^Department of Environmental Medicine and Public Health, Icahn School of Medicine at Mount Sinai, New York, NY, United States; ^7^Department of Biochemistry, Medical University of Gdansk, Gdansk, Poland; ^8^Toronto General Hospital Research Institute (TGHRI), University Health Network, Toronto, ON, Canada; ^9^Ted Roger’s Center for Heart Research, University Health Network, Toronto, ON, Canada

**Keywords:** heart transplantation (HTx), *ex situ* heart perfusion, immunity response, solid phase microextraction (SPME), metabolomics, lipidomics

## Abstract

*Ex situ* heart perfusion (ESHP) was developed to preserve and evaluate donated hearts in a perfused beating state. However, myocardial function declines during ESHP, which limits the duration of perfusion and the potential to expand the donor pool. In this research, we combine a novel, minimally-invasive sampling approach with comparative global metabolite profiling to evaluate changes in the metabolomic patterns associated with declines in myocardial function during ESHP. Biocompatible solid-phase microextraction (SPME) microprobes serving as chemical biopsy were used to sample heart tissue and perfusate in a translational porcine ESHP model and a small cohort of clinical cases. In addition, six core-needle biopsies of the left ventricular wall were collected to compare the performance of our SPME sampling method against that of traditional tissue-collection. Our state-of-the-art metabolomics platform allowed us to identify a large number of significantly altered metabolites and lipid species that presented comparable profile of alterations to conventional biopsies. However, significant discrepancies in the pool of identified analytes using two sampling methods (SPME vs. biopsy) were also identified concerning mainly compounds susceptible to dynamic biotransformation and most likely being a result of low-invasive nature of SPME. Overall, our results revealed striking metabolic alterations during prolonged 8h-ESHP associated with uncontrolled inflammation not counterbalanced by resolution, endothelial injury, accelerated mitochondrial oxidative stress, the disruption of mitochondrial bioenergetics, and the accumulation of harmful lipid species. In conclusion, the combination of perfusion parameters and metabolomics can uncover various mechanisms of organ injury and recovery, which can help differentiate between donor hearts that are transplantable from those that should be discarded.

## Introduction

Heart transplantation (HTx) is the “gold-standard” treatment for eligible patients with advanced heart failure, but its use continues to be limited by organ availability (1, 2). Hearts are typically procured following the neurological determination of death (NDD); however, less than 40% of hearts are deemed suitable for transplantation, with the remainder being discarded based on clinical parameters that strongly predict primary graft dysfunction (3). Two notable options for expanding the donor organ pool are the use of 1) extended-criteria donation (ECD), and 2) donation after circulatory death (DCD) (3). The first approach involves using hearts with higher-risk features, such as advanced donor age, left ventricular hypertrophy, or longer predicted ischemic time, while the latter approach involves procuring hearts following donor cardiocirculatory arrest. DCD organs are invariably subjected to a period of unprotected ischemia, which can potentially cause irreversible tissue damage (4). With either option, the ability to functionally evaluate the heart and prevent further ischemia-reperfusion injury (IRI) during transportation is critical to ensuring optimal outcomes.

*Ex Situ* Heart Perfusion (ESHP) is an alternative strategy to traditional static cold storage that has been garnering increasing clinical interest for the preservation and assessment of donor hearts prior to transplantation (5, 6). Normothermic ESHP maintains aerobic metabolism and limits cold ischemia by providing oxygenated, nutrient-enriched perfusate to the heart during preservation (7). In addition, normothermic ESHP also offers the unique opportunity to provide supplemental pharmacological agents for protective ischemic postconditioning, which can potentially enable the recovery of dysfunctional hearts (8, 9).

Numerous pre-clinical transplantation studies have utilized ESHP to preserve donor hearts, with results demonstrating improved functional recovery and injury profiles compared to cold storage (3). Clinically, the use of normothermic ESHP has yielded excellent results, enabling the use of ECD and DCD hearts and expanding the donor pool by over 30% (10). Despite these successful applications, many questions regarding the optimization of ESHP remain unanswered. For instance, alterations in myocardial metabolic conditions and substrate utilization may contribute to the significant decline in myocardial function during prolonged ESHP, and may therefore represent an avenue for improving donor heart preservation (11).

Metabolomics involves studying the downstream products of multiple physiological and pathological processes, and may therefore be able to provide a unique broad-spectrum perspective of substrate utilization in donor hearts during ESHP (12–14). Furthermore, the metabolomic signatures of machine-perfused hearts remain unexplored, and these signatures could play a key role in optimizing organ preservation and preventing functional decline.

Solid-phase microextraction (SPME) has emerged as a novel, minimally-invasive sample-preparation approach that enables the extraction/analysis of a broad spectrum of metabolites in a variety of matrices without requiring the removal of tissue samples (15, 16). In SPME, a chemical biopsy microprobe approximately the size of an acupuncture needle (200 µm in diameter) coated with a biocompatible polymeric extraction phase (40 µm thickness) is inserted into the tissue until the coating is fully immersed, followed by a short extraction period of only the small compounds, while leaving other components in tissue (17). Notably, the SPME methodology allows for repeated measurements, which would not be feasible using conventional methods. This solvent-less extraction technique, which is based on the equilibration between the sample and the sorbent coating on the probe, has already been proven to offer several additional advantages over traditional extraction methods, including the ability to define a pool of metabolites subjected to a rapid and extensive conversion in dynamic biochemical processes (16). When coupled with liquid chromatography and highly sensitive mass detectors, SPME enables the profiling of specific metabolic pathways or global metabolites, thereby providing an instant snapshot of the tissue biology at a fixed time (17).

In this paper, we document the comprehensive metabolomic profiling of *ex situ* perfused hearts. Specifically, we seek to: 1) evaluate the potential of SPME probes for non-destructive metabolite extraction over the course of ESHP; and 2) determine the metabolic profile of the heart during perfusion, and how this relates to functional decline.

## Materials and Methods

### Study Protocol

**Figure 1:**
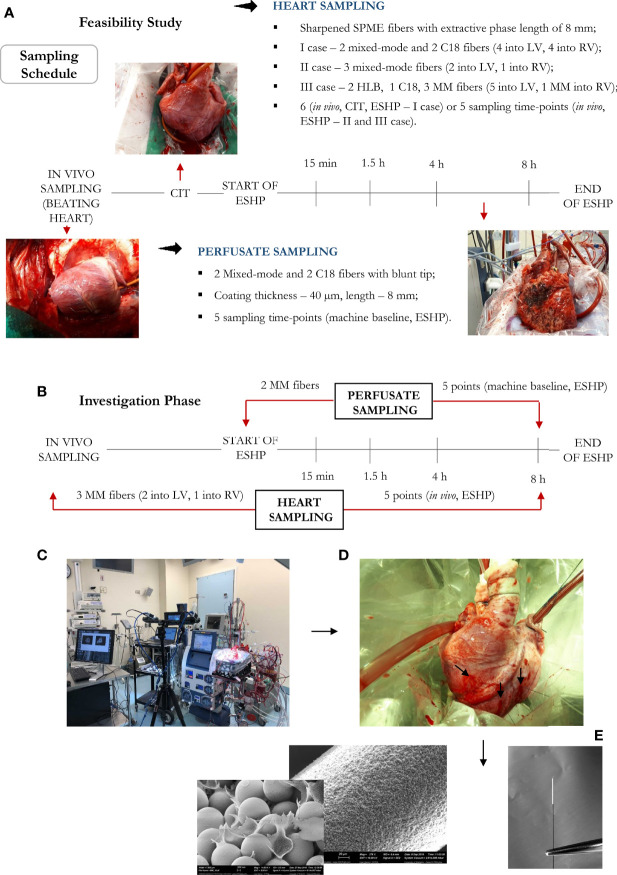
Bio-SPME sampling of porcine hearts during *ex situ* heart perfusion (ESHP). Time schedule of heart and plasma/perfusate sampling for the **(A)** feasibility and **(B)** investigation phases. Photograph of the ESHP circuit **(C)** together with solid-phase microextraction (SPME) microprobes applied for *in*/*ex vivo* and non-destructive sampling of small-molecular-weight molecules **(D)**. Insertion of a microprobe the size of an acupuncture needle (0.28 mm diameter) into cardiac tissue such that its entire 8 mm coating is immersed. Enlarged images of SPME fibers with a sharpened tip were presented **(E)**. The SPME probe enables minimally-invasive/non-destructive sampling, and is manufactured from fully biocompatible materials.

Eight porcine hearts were evaluated over 8 hours of ESHP. The first 3 hearts were used to determine the optimal protocol for metabolomic assessment (feasibility phase), while the remaining 5 hearts were used to determine the most pronounced metabolic alterations and their association with the progressive decline in myocardial function during ESHP (study phase). Finally, 2 human hearts were procured from NDD donors and evaluated over 8 hours of ESHP; these hearts had been rejected for transplantation by all transplant centers and had received consent for use in research. The study protocol is summarized in **Figure 1**.

The experimental protocol was approved by the Institutional Review Board (IRB) at the University Health Network (UHN; Toronto, ON, Canada) and University of Waterloo’s Research Ethics Boards (# 40573), and all procedures conformed to the NIH guidelines (Guide for the Care and Use of Laboratory Animals). This study also received approval from the Province of Ontario’s organ procurement organization and was conducted in accordance with the 1964 Declaration of Helsinki.

### Donor Heart Procurement

Hearts from male Yorkshire pigs (40 ± 5 kg) were procured according to a standard NDD protocol, which has been previously described (5, 18, 19). In brief, the animals were premedicated with intramuscular Midazolam (0.3 mg/kg) and Ketamine (20 mg/kg), and anesthesia was induced and maintained *via* inhalational isoflurane administered through an endotracheal tube (end tidal concentration:1-3%). An arterial line was inserted through one of the carotid arteries, a central venous line was introduced into a jugular vein, and a pulmonary artery catheter was inserted *via* the other jugular vein and directed beyond the pulmonary artery bifurcation. A median sternotomy was performed to expose the heart and great vessels. Umbilical tape was then placed around the inferior vena cava, and a pressure-volume conductance catheter (Millar Instruments Inc, USA) was inserted into the left ventricle *via* an apical ventriculotomy. Systemic anticoagulation was achieved with an injection of 30,000 U heparin. Following a baseline evaluation, a cardioplegia cannula was placed in the ascending aorta, and an 18F venous cannula was placed in the right atrium. We collected 1.5 L of whole blood into an autotransfusion system (Frensenius Kabi C.A.T.S., Terumo, USA) in order to isolate the red blood cells (RBC). Simultaneously, the aorta was cross-clamped, and the heart was arrested using 1 L of histidine-ketoglutarate-tryptophan (HTK) at 4°C. Once arrested, the heart was excised and placed in ice-cold HTK for 1 hour while being cannulated for ESHP.

Both human hearts were procured using a similar protocol. The first human heart was acquired from a 57-year-old male who had suffered an acute ischemic stroke and was subsequently declared brain dead. This donor’s comorbidities included type I diabetes, smoking, renal failure, and hypertension. This donor’s heart had normal biventricular function with mild concentric left ventricular hypertrophy. The second heart was acquired from a 67-year-old male who had suffered a severe traumatic brain injury and was declared brain dead. This donor’s comorbidities included a previous stroke, hypertension, and smoking. No pre-procurement cardiac testing was performed for this heart. The two human hearts were flushed and preserved for five and twelve hours, respectively.

### *Ex Situ* Heart Perfusion

We used a custom ESHP system, which has been described in previous works (5, 19). In brief, the system was primed with 500 mL of STEEN Solution™ (XVIVO Perfusion, USA), 500 mL of SOM-TRN-001 (Somahlution, USA), 1 mg/kg of mannitol, 500 mg of methylprednisolone, 10,000 U of heparin, and 1 g of cefazolin. A sufficient volume of RBCs was added to achieve a hematocrit of 15%. Based on previous reports of pro-inflammatory and oxidant effects of a whole blood-based perfusate (20) and in an attempt to avoid the use of donor plasma given the known catecholamine storm following both donation after neurologically determined death and circulatory death, our group sought to optimize the perfusate composition with plasma substitutes. We opted for this setup based on previous studies from our group comparing whole blood-based perfusate with STEEN and SOM-TRN-001 (5, 19, 21). STEEN Solution™ is a buffered extracellular solution with an optimal colloid osmotic pressure developed specifically for *ex situ* perfusion of lungs (22). Its primary components - Dextran-40 and albumin - function synergistically as oncotic agents, helping to keep the water in the intravascular compartment and therefore decreasing interstitial edema. Dextran-40 also acts as a scavenger that coats and protects the endothelium from subsequent excessive leukocyte interaction during reperfusion. The glucose component is designed to provide a metabolic substrate for aerobic metabolism during prolonged ischemia. These have been shown to both protect the endothelium and decrease edema during machine perfusion (23). SOM-TRN-001 is a novel cardioprotective solution that combines various energy substrates, metabolic modulators, free radical scavengers, antioxidants, ammonia chelators, nitric oxide synthase substrates, intracellular and extracellular hydrogen chelators, and a physiological concentration of calcium to meet the energy requirements of cardiomyocytes and coronary endothelium (24). Together, both STEEN and SOM-TRN-001 possess interesting compositions that could improve heart preservation during ESHP. The former, by actively counteracting the positive hydrostatic pressure of machine perfusion and decreasing interstitial edema; the latter, by decreasing reperfusion injury and priming the organ for metabolic and functional recovery. Additionally, contrary to other preservation solutions, neither exert cardioplegic effects, making them ideal candidates for ESHP as a plasma substitute. Calcium chloride (10%), sodium bicarbonate (8.4%), and dextrose (50%) were added to correct the calcium (1.1-1.3 mmol/L), glucose (5-10 mmol/L), and bicarbonate (24-30 mmol/L) concentrations, respectively, and dobutamine (5 µg/min), insulin (5 U/h), and nitroglycerin (1 µg/kg/min) were infused continuously.

The hearts were mounted on the ESHP system following 1 hour of cold storage. Once de-airing was complete, retrograde aortic root perfusion was started at 40 mmHg, and the hearts were rewarmed to 37°C over a 30-minute period by increasing the temperature of the perfusate. Arterial and venous perfusate samples were collected every hour to measure pH, lactate levels, hemoglobin, and hematocrit, as well as to evaluate gas exchange using a point-of-care blood gas analyzer (RAPIDPoint^®^ 500 Blood Gas Systems, Siemens). The left ventricle was loaded to a left atrial pressure of 8 mmHg at 2, 4, and 8 hours for functional assessment, and metabolomic data was collected at 15 minutes, 1.5, 4, and 8 hours of perfusion.

### Functional Assessment of the Heart

The left ventricle’s functional parameters were measured using a conductance catheter (Ventri-Cath-507S, Millar Inc, Houston, TX) that had been calibrated according to the manufacturer’s instructions to record pressure-volume loops and relationships using IOX v1.8.9.13 software (EMKA Technologies Inc., Falls Church, VA). Volume-dependent measurements (i.e., stroke work, maximum and minimum rate of developed pressure) were collected under steady loading conditions aimed at maintaining a left atrial pressure of 8 mmHg, while volume-independent measurements (i.e., preload recruitable stroke work) were collected by intermittently decreasing left atrial loading (19).

### Untargeted Metabolomic and Lipidomic Analysis

#### Bio-SPME Probes

This study used SPME fibers (Supelco, Sigma Aldrich, USA) that had been developed using 200 µm nitinol wires, with an extraction phase length of 8 mm and a coating thickness of 40 µm (**Figure 1**). Two extractive phases were selected for use in this work: 1) mixed-mode particles (MM – C8/SCX)—a combination of a cation exchanger and octyl functionalized silicate—were selected for general metabolomic investigations, and 2) octadecyl functionalized silicate particles (C18) were selected for the lipidomic investigations. Additionally, hydrophilic-lipophilic balance (HLB) particles were investigated alongside the C8-SCX and C18 coatings as an alternative extraction phase, as prior findings suggest they can offer improved affinity towards specific classes of polar metabolites (25).

#### Extraction Protocol

Metabolomic and lipidomic investigations were performed by inserting the SPME probes into the myocardium (LV and RV) and immersing them in the perfusate. Extractions were carried out for 20 minutes, with the probes being subsequently rinsed manually in water for 5 seconds and snap frozen. The experimental protocols for the feasibility and study phases are illustrated in **Figure 1**. The optimization framework for early-stage design included evaluations of the following parameters: 1) the selectivity/performance of SPME coatings containing: C8 and benzenesulfonic acid (C8/SCX), C18 or HLB functionalities; 2) the time-course of sample collection; 3) the number of samples taken at each sampling time; and 4) the instrumental and data acquisition conditions required to capture and identify a broad range of metabolites. The most suitable coating chemistry was selected based on number of analytes extracted with the highest efficiency, reproducibility of the extracted amounts, and the coating’s commercial availability. Finally, we collected core-needle biopsies of the left ventricular (LV) free wall *in vivo* and at the end of the 8-hour perfusion, which were used to compare the performance of our SPME sampling method against that of traditional biopsies. A detailed method of processing tissue for metabolomic examination has been described elsewhere (26). Briefly, the muscle samples were homogenized with prechilled MeOH/H_2_O (1:1, v/v) using a Precellys bead beater (40 s per cycle, 6,500 Hz speed for each cycle) in 10 volumes of solvent against weight of the tissue (v/w). Samples were centrifuged at 15,000 rpm for 15 min at 4°C and the supernatant was retained. Next, the prechilled solution of IPA/H_2_O (1:1, v/v) was added to the tissue pellet. Homogenization of samples was repeated using a bead beater with the same parameters as those indicated above. The mixture was centrifuged at 15,000 rpm for 15 min and the supernatant combined with that collected in the previous step. The pooled extracts were dried in speed-vac concentrator before reconstitution for LC/MS analysis (50:50, ACN/H_2_O, v/v).

#### Metabolomic and Lipidomic Investigations

Prior to instrumental analysis, the MM and C18 probes were desorbed in 60 µL of ACN/H_2_0 (8:2, v/v) and 70 µL of MeOH/IPA/H_2_O (45:45:10, v/v/v), respectively, using mechanical agitation at 1500 rpm for 60 min. The samples collected were directly injected into the analytical system. Additionally, the extracts obtained from desorbing the analytes from the MM probes in the early optimization phase of the study were split into two separate fractions, which were subjected to independent instrumental evaluations using two distinct chromatographic modes: pentafluorophenyl (PFP)-based and hydrophilic interaction chromatography (HILIC) mode. In order to monitor performance, a quality control (QC) sample was prepared as a pooled mixture of sample aliquots and analyzed at 10-sample intervals throughout the run.

Liquid chromatography/mass spectrometry (LC/MS) analyses were performed using a Vanquish UHPLC system (Thermo Scientific) interfaced with a Thermo Scientific Exactive Orbitrap high-resolution accurate mass spectrometer *via* an electrospray ionization (ESI) source. The metabolomic investigations were conducted using a Discovery HS F5 (100 x 2.1 mm, 3 µm particle size, Supelco) column maintained at 25°C, and an SeQuant^®^ ZIC^®^-HILIC (3.5 µm, 100 x 2.1 mm, Millipore) column maintained at 40°C. The binary mobile phases consisted of deionized water (A) and ACN (B), with 0.1% formic acid (ESI+ mode) or 1 mM acetic acid (ESI- mode). The conditions employed for chromatographic separations and relevant mass detection have been described in detail elsewhere (27). For the lipidomic investigations, chromatographic separation was performed at 55°C with an XSelect CSH C18 column (2.1 × 75 mm, 3.5 µm) using a two-solvent system composed of solvent A [40:60 MeOH/H_2_O with 10 mM ammonium acetate and 1 mM acetic acid (positive mode) or with 0.02% acetic acid (negative mode)] and solvent B [90:10 IPA:MeOH with 10 mM ammonium acetate and 1 mM acetic acid (positive mode) or with 0.02% acetic acid (negative mode)]. A detailed description of this method may be found in (28). Finally, the experimental sample injection order was randomized, with an injection volume of 10 µL being used for all analyses.

### Data Handling and Statistical Analysis

Functional data were reported as median values (interquartile range) and compared using the Kruskal-Wallis test, while *post-hoc* analyses were performed using Dunn’s multiple comparisons test. Analyses were performed using SPSS 24.0 (IBM, USA), with a p-value<0.05 being considered significant.

ProteoWizard MS Convert 3.0.2 was used to convert the raw LC/MS data to mzXML files (29), which were subsequently processed with XCMS Online for peak detection, alignment, and isotope annotation (30). Data processing yielded a multi-dimensional peak table, which included accurate mass-to-charge ratios (m/z), retention times (RT), and peak areas. The generated feature (i.e., a molecular entity with a unique m/z and RT value) list, which comprised their integrated intensities, was further subjected to principal component analysis (PCA) to visualize the general structure of each data set, and to identify sub-groups and any potential outliers within the data. Furthermore, data log transformation and Pareto scaling were utilized to adjust for potential differences among the study samples, and to make individual features more comparable. Supervised partial least squares discriminant analysis (PLS-DA) was then applied to maximize variation among the study groups, and to determine which metabolites contributed to it. The quality of the generated PLS-DA models was validated based on two parameters: the model’s goodness-of-fit (R2), and its prediction ability as indicated by the cumulative Q2 value (Q2cum). Each metabolite’s influence on the classification (into specific groups) was calculated based on its variable influence on projection (VIP). In this study, only metabolites with VIP values ≥1.5 were selected for further investigation (e.g. pathway analysis), which was conducted using free web based MetaboAnalyst 5.0 software (31). Finally, ANOVA testing with Fisher’s *post-hoc* analysis and false discovery rate (FDR) analysis were applied to datasets obtained across different time points. The ANOVA test results (P < 0.05 and FDR (q) < 0.05) were combined with the VIP values of the first principal component in order to search for distinct metabolites. Metabolites were identified by conducting database searches against their accurate masses, as well as *via* fragmentation pattern analysis and retention-time comparisons when authentic standards were available. Computational annotation of the features was performed using the xMSannotator v1.3.2 package in R in order to find matches in the HMDB (Human Metabolome Database), LIPID MAPS, or KEGG (Kyoto Encyclopedia of Genes and Genomes) databases (32). xMSannotator – the clustering algorithm uses a multistep strategy for annotation, including intensity profiles, retention time characteristics, mass defect, and isotope/adduct patterns with confidence level assignment to annotation results as a final outcome. In this context, each chemical entity was categorized as no confidence (0), low confidence (1), medium confidence, (2) or high confidence (3). For further metabolomics investigations, including pathway enrichment analysis, only unique and multiple features with medium-to-high confidence annotations were selected. For high-confidence matches, the following requirements were satisfied: (i) a non-zero score for database matching, (ii) presence of required adducts/forms specified by the user (i.e. M+H or M-H for positive and negative modes respectively), (iii) N, O, P, S/C ratio checks as well as hydrogen/carbon ratio check, (iv) abundance ratio checks for isotopes, multimers, and multiply charged adducts with respect to the singly charged adducts/ions. In turn, medium confidence was assigned based on the pathway level correlation (for unique features defined by the algorithm).

## Results

### Protocol Feasibility

In the early feasibility phase, the performance of various SPME extractive phases, sampling schedules, and analytical conditions were examined in order to identify the optimal strategy for analysis.

The HLB coating yielded the best results of the three tested coatings, providing broader metabolite coverage and greater intensities for significant detected features (**Supplementary Figures 1–10**). Compared to the C18 coating, the MM coating exhibited superior recovery for the majority of polar and moderately hydrophobic compounds. In addition, the MM coating also provided a pool of metabolites that showed more dynamic fluctuation throughout ESHP, indicating its ability to provide coverage for a more informative subset of compounds. Since HLB fibers are not yet commercially available, and their further implementation in the routine metabolomic workflow would be cumbersome, MM fibers offer a good compromise between suitability for high-throughput assays and the right balance of metabolite coverage (**Supplementary Figures 1–10**).

Six sampling time points were selected for investigation: 1) sampling from a beating heart; 2) under cold ischemic time (CIT); and 3) sampling the heart at four different time intervals over an 8 h period of *ex situ* perfusion. Although cellular metabolism is reduced by approximately 10 times during cold static storage, low-level biochemical processes continue to take place, which may lead to organ injury upon further reperfusion (33); therefore, to minimize the impact of any additional factors that may account for IRI and consequent graft dysfunction, samples were only collected during CIT in the case of the 1st pig, with this sampling point being omitted during further investigations. On the other hand, the first two sampling time points were set at 15 min and 1.5 h of ESHP, as metabolic processes are thought to be most dynamic during the immediate post-reperfusion period. The other timepoints were set at larger intervals, taking place at 4 h and 8 h (**Figure 1**). Additionally, to limit any possible organ stress associated with the insertion of SPME fibers, the number of microprobes was reduced to the minimum amount that would permit the extraction of sufficient biological information (i.e., two probes in the LV, one in the RV). Overall, three bio-SPME probes with MM coating were employed to track cardiac metabolic alterations across five time intervals. In addition, the perfusate was sampled in parallel with heart sampling events using two MM fibers per event (**Figure 1B** and **Supplementary Figures 1–10**).

**Figure 2:**
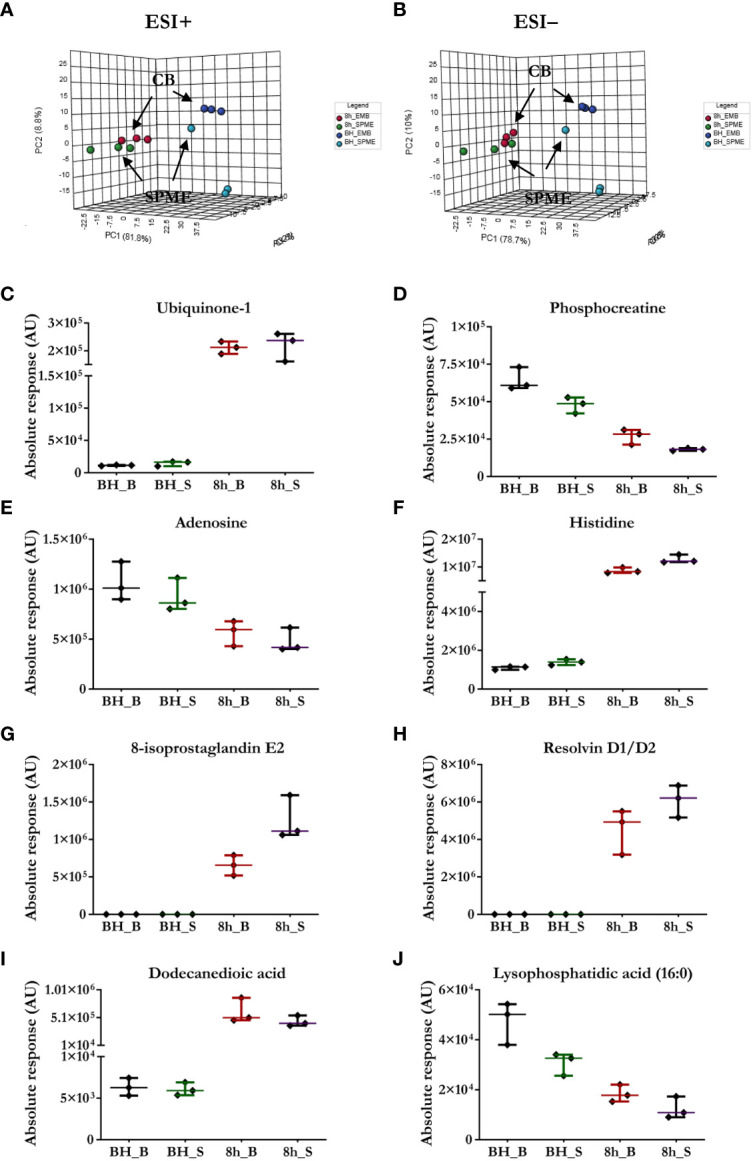
Comparison of the performance of our newly developed sampling method and a standard biopsy-collection method. **(A, B)** The principal component analysis (PCA) for metabolic features detected in SPME or biopsy samples (CB) implies relative agreement between both approaches. Light blue—*in vivo* LV (left ventricle) SPME samples; dark blue—*in vivo* LV biopsy samples; green—SPME samples taken at 8 h of ESHP; red—biopsy samples collected at 8 h of ESHP. **(C–J)** Scatter plots showing relative intensities for selected metabolic features attained using the two sampling methods. Each dot in the scatter plots represents an individual metabolite/lipid in each sample. BH_B/S—*in vivo* LV biopsy/SPME samples; 8h_B/S—8 h ESHP biopsy/SPME samples.

**Figure 3:**
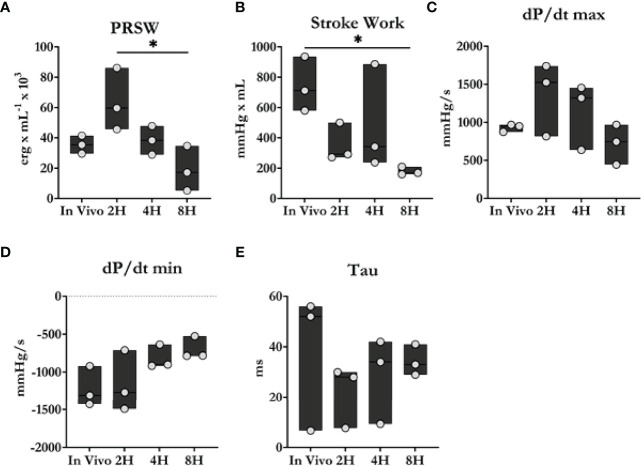
Cardiac contractility comparison between *in vivo* and *ex situ* conditions. **(A)** Preload recruitable stroke work (PRSW). **(B)** Stroke work. **(C, D)** Maximum and minimum dP/dt. **(E)** Time constant of relaxation. Overall, heart function progressively declined during the 8 h perfusion period. Data were reported as median values and compared using the Kruskal-Wallis test. *Post-hoc* analyses were performed using Dunn’s multiple comparisons test. *P < 0.05.

**Figure 4:**
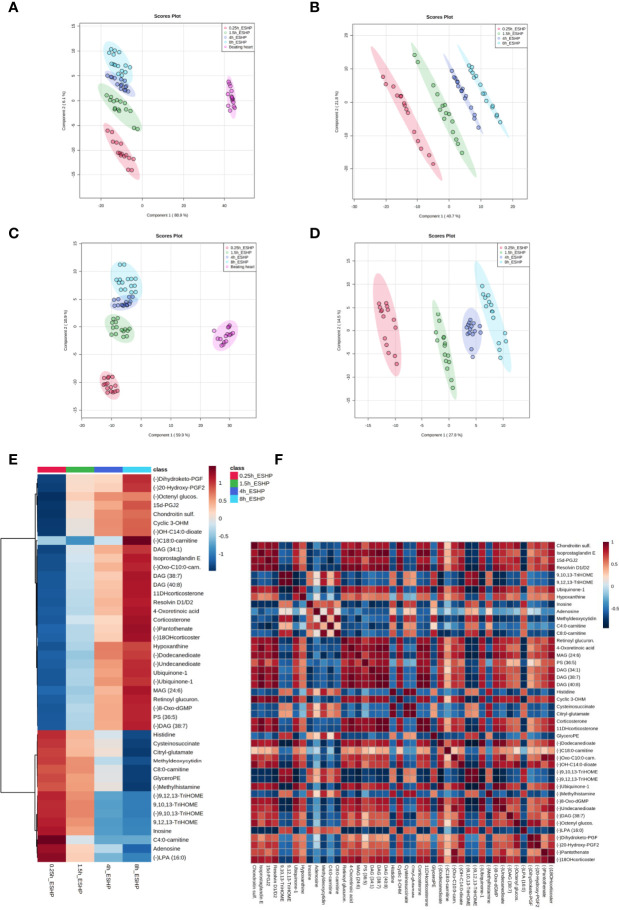
Dynamic changes in metabolomic pattern of *ex situ* perfused porcine hearts. Partial least square discriminant analysis (PLS-DA) models comparing LC/MS metabolomic profiles among five **(A, C)** and four **(B, D)** conditions corresponding to relevant sampling time points during *in vivo* and *ex situ* heart perfusion, as well as heatmap and correlation analysis for the most discriminative metabolites **(E, F)**. The presented score plots were created for metabolic features detected in ESI+ **(A, B)** and ESI- **(C**, **D)**/PFP-based mode. The degree of variance (reflecting the strength of the constructed model) is shown in parentheses on each axis. The different groups of samples are presented using different colors [each dot represents an individual sample (one biological replicate)]: pink—samples collected *in vivo* (from beating heart); red—samples collected 15 min after commencing ESHP; green—samples collected 1.5 h after commencing ESHP; dark blue—samples collected 4 h after commencing ESHP; light blue—samples collected 8 h after commencing ESHP. Circles represent 95% confidence intervals. Additionally, the robustness of the PLS-DA models was validated *via* leave-one-out cross-validation (LOOCV) and calculating relevant Q2 and R2 values. The 5-component cross-validation measures for R2Xcum of 0.998 (ESHP/ESI+) and 0.998 (ESHP/ESI-), and the Q2cum values of 0.997 (ESHP/ESI+) and 0.995 (ESHP/ESI-) are excellent and provide good fit to the data and high predictive ability. **(E)** Heatmap analysis highlighting metabolites/lipid species showing the largest change in cardiac tissue between 15 min and 8 h of *ex situ* heart perfusion. **(F)** Correlation plot showing significant correlations between cardiac metabolite levels altered throughout ESHP (Pearson’s R). (–) refers to negative ionization mode. The labels on the x and y-axis on the heatmaps are frequently presented as abbreviations (due to limited space). Dihydroketo-PGF2 – 8-iso-13,14-dihydro-15-keto-PGF2a; 20-Hydroxy-PGF2 – 20-Hydroxy-PGF2a; Octenyl glucos. – 1-Octen-3-yl glucoside; 15d-PGJ2 – 15-Deoxy-d-12,14-PGJ2; cyclic 3OHM – Cyclic 3-Hydroxymelatonin; OH-C14:0-dioate – 3-Hydroxytetradecanedioic acid; C18:0-carnitine – Stearoylcarnitine; Oxo-C10:0-carn – 6-Keto-decanoylcarnitine; 11-DHcorticosterone – 11-Dehydrocorticosterone; 18-OHcortico-sterone – 18-Hydroxycorticosterone; C8:0-carnitine – Octanoylcarnitine; C4:0-carnitine – Butyrylcarnitine; TriHOME – Trihydroxyoctadecenoic acid.

**Figure 5:**
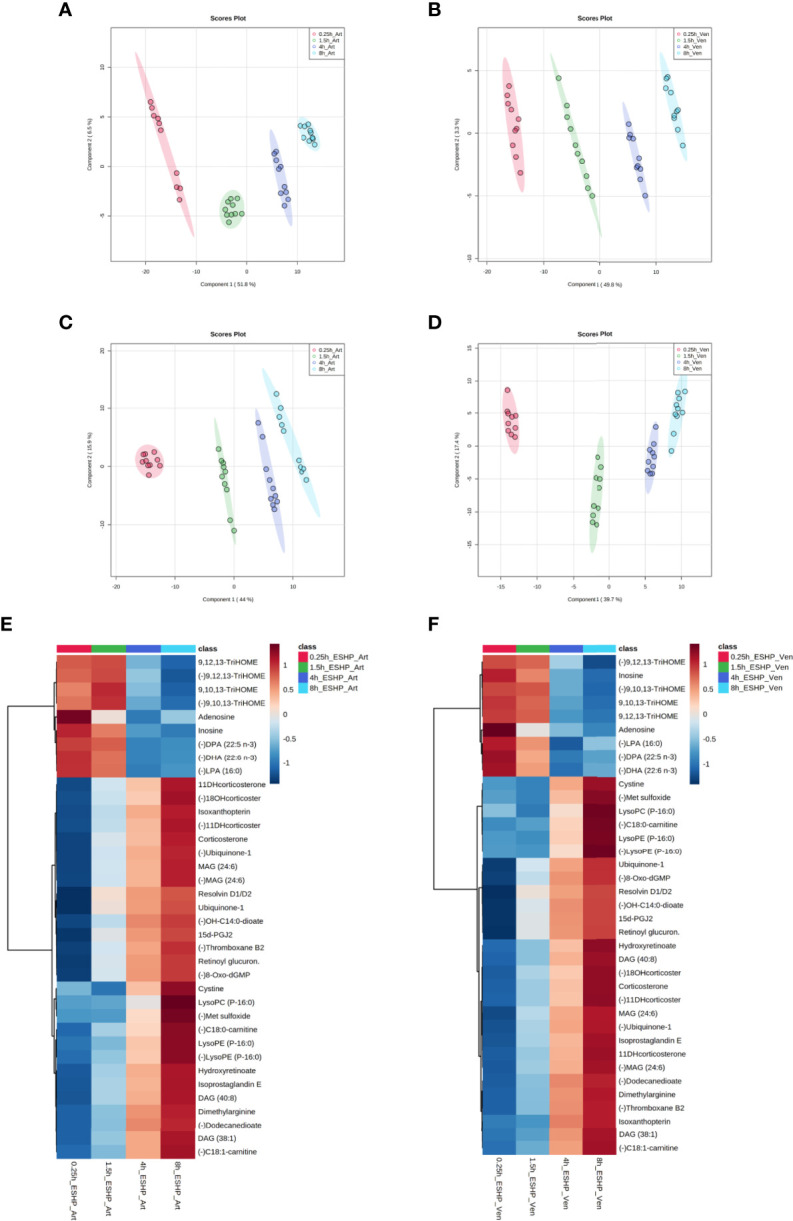
Metabolic alterations in perfusate samples during porcine ESHP. PLS-DA score plots depicting changes in metabolomic patterns throughout *ex situ* heart perfusion at the time of perfusate sampling **(A–D)**, and color-coded maps illustrating the average abundance of the most altered metabolic features when the 8 h sampling time-point was compared against the 15 min, 1.5 h, and 4 h time points **(E, F)**. The models were created for metabolic features detected in ESI+ **(A, B)** and ESI- **(C, D)**/PFP-based mode. Red—arterial/venous perfusate samples taken at 15 min of ESHP; green—arterial/venous perfusate samples taken at 1.5 h of ESHP; dark blue—arterial/venous perfusate samples taken at 4 h of ESHP; light blue—arterial/venous perfusate samples taken at 8 h of ESHP. The predictive ability of the generated models (during PLS-DA analysis) was verified *via* LOOCV, and strong predictive performance was confirmed, with Q2~0.9 (for 5-component cross-validation: 0.997 and 0.998 for ESI+/Art and ESI+/Ven samples, respectively, and 0.998 and 0.997 for ESI-/Art and ESI-/Ven samples, respectively). **(E, F)** Heat maps of metabolomic differences between perfusate samples taken at various time points during ESHP. (–) refers to negative ionization mode. TriHOME – Trihydroxyoctadecenoic acid; DPA (22:5 n-3) – Docosapentaenoic acid; DHA (22:6 n-3) – Docosahexaenoic acid; 11-DHcorticosterone – 11-Dehydrocorticosterone; 18-OHcorticosterone – 18-Hydroxycortico-sterone; OH-C14:0-dioate – 3-Hydroxytetradecanedioic acid; 15d-PGJ2 – 15-Deoxy-d-12,14-PGJ2; C18:0-carnitine – Stearoylcarnitine; C18:1-carnitine – Octadecenylcarnitine.

**Figure 6:**
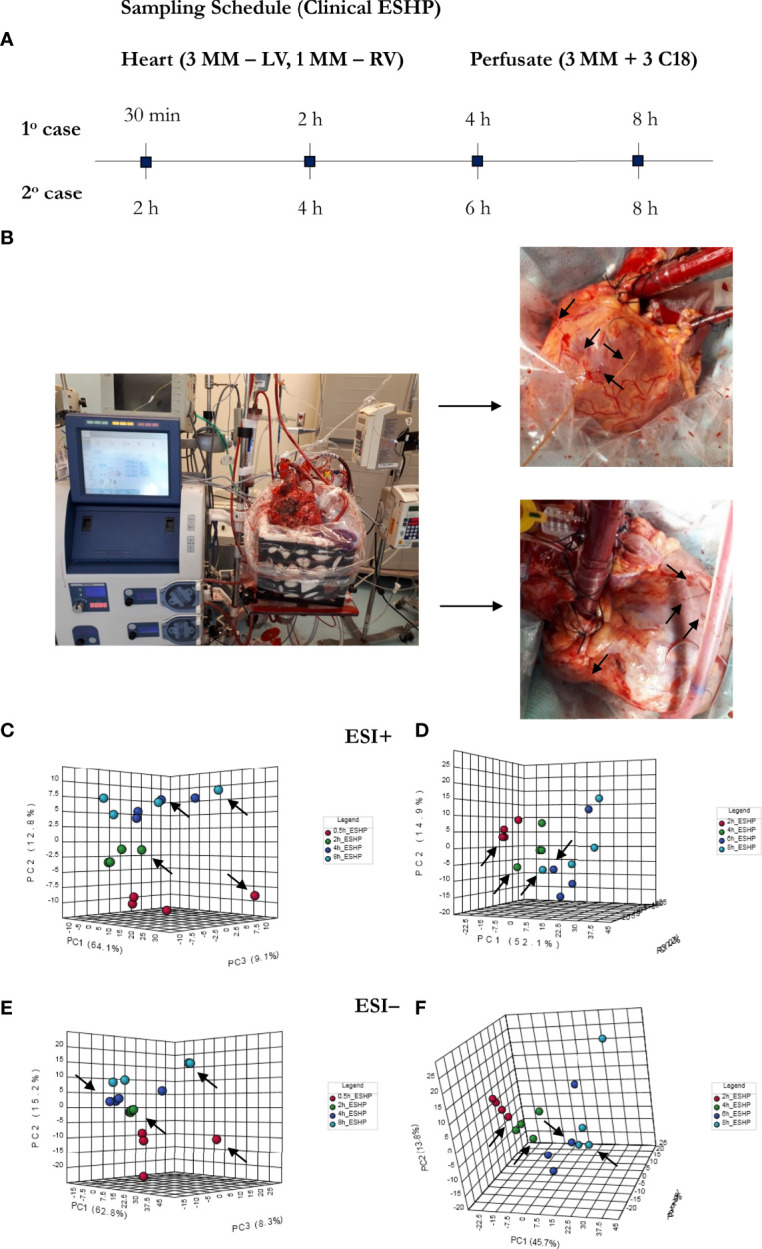
Bio-SPME sampling of human hearts during ESHP. **(A)** Time schedule of heart and perfusate sampling. **(B)** Three Bio-SPME probes inserted into the LV (left ventricle) to a depth of 8 mm (full coating length), and one microprobe inserted into the RV (right ventricle). **(C–F)** PCA (principal component analysis) score plots for metabolites detected in human heart samples collected *ex vivo via* SPME. Relevant plots were generated for metabolic features detected in ESI+ **(C, D)** and ESI- **(E, F)**/PFP-based mode. The different groups of samples (associated with duration of perfusion) are distinguished using different colors. For clinical case 1: red—samples taken at 30 min of ESHP; green—samples taken at 2 h of ESHP; dark blue—samples taken at 4 h of ESHP; light blue—samples taken at 8 h of ESHP. For clinical case 2: red—samples taken at 2 h of ESHP; green—samples taken at 4 h of ESHP; dark blue—samples taken at 6 h of ESHP; light blue—samples taken at 8 h of ESHP. The arrows depict samples collected from the RV.

**Figure 7:**
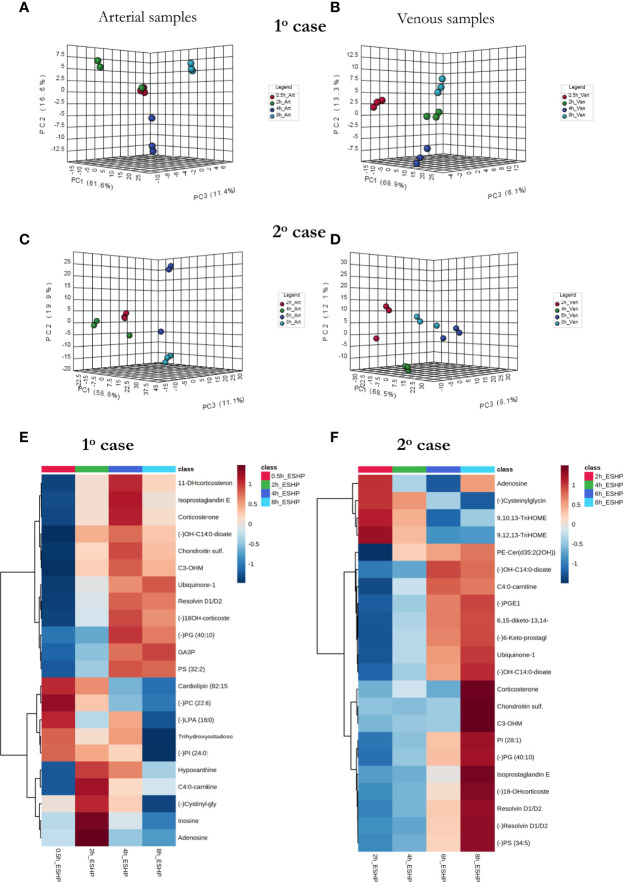
The comprehensive picture of metabolomic alterations identified in perfusate and cardiac samples during clinical ESHP. **(A–D)** PCA score plots for metabolites detected in perfusate samples collected during clinical ESHP, and **(E, F)** heat maps depicting the most altered metabolites in human hearts over the course of ESHP. The plots were generated for metabolic features detected in 1^st^ case **(A, B)** and 2^nd^ case **(C, D)**. For clinical case 1: red—arterial/venous perfusate samples taken at 30 min of ESHP; green—arterial/venous perfusate samples taken at 2 h of ESHP; dark blue—arterial/venous perfusate samples taken at 4 h of ESHP; light blue—arterial/venous perfusate samples taken at 8 h of ESHP. For clinical case 2: red—arterial/venous perfusate samples taken at 2 h of ESHP; green—arterial/venous perfusate samples taken at 4 h of ESHP; dark blue—arterial/venous perfusate samples taken at 6 h of ESHP; light blue—arterial/venous perfusate samples taken at 8 h of ESHP. (–) refers to negative ionization mode. 11-DHcorticosterone – 11-Dehydrocorticosterone; OH-C14:0-dioate – 3-Hydroxytetradecane-dioic acid; C3-OHM – Cyclic 3-Hydroxymelatonin; 18-OHcorticosterone – 18-Hydroxycortico-sterone; GA3P – Glyceraldehyde-3-phosphate; C4:0-carnitine – Butyrylcarnitine; Cysteinylglycin. – L-Cysteinylglycine disulfide; 6,15-diketo-13,14. – 6,15-diketo-13,14-dihydro-prostaglandin F1α; 6-Keto-prostagl. – 6-Keto-prostaglandin F1α.

Three chromatographic modes—pentafluorophenyl (PFP)-bonded phase, HILIC, and C18-bonded phase—were tested with the aim of expanding metabolite coverage. Although PFP and C18 offered complementary reversed-phase selectivity, PFP phase outperformed C18 and HILIC in terms of the number of metabolic features it retained (see **Figures 1–10** in the **Supplementary File**). However, PFP provided worse coverage than HILIC in holding highly polar metabolites. Nonetheless, the PFP phase provided the best results in respect to the capability to capture the broadest range of analytes, particularly with polar and moderately hydrophobic properties constituting major fraction of metabolic intermediates engaged in energy generation thus was selected for use in further metabolomic investigations. The numerical values describing the performance of each of the assay applied (expressed as the number of features detected) for each of three cases involved, diverse matrixes investigated (heart tissue, perfusate), two ionization modes have been presented in detail in **Supplemental Information** (**Supplementary Figures 1–10**). Furthermore, the representative HPLC-MS base peak chromatograms (BPCs) for these analytical assays have been shown in **Supplementary Figures 11–13**.

The suitability of SPME microprobes for assessing organ preservation was confirmed by comparing this approach to LC/MS assessments of cardiac biopsies. LC/MS data sets obtained *via* positive and negative ion mode for two sampling intervals were subjected to the same analysis, with significant clusters being observed in the LC/MS spectral features between the groups: *in vivo* SPME vs. biopsies, and 8 h-ESHP SPME vs. biopsies (**Figure 2**). Notably, samples matching based on their location of collection was characterized by a greater degree of tight clustering, reflecting a more convergent metabolic pattern among the investigated samples, and indicating that SPME is capable of capturing the comparable dynamics of matrix metabolites (when compared with standard biopsy collection). However, some significant discrepancies between the two methods were found, which were likely due to various tendencies to preserve the intact metabolome, the nature of sample processing, and a lack of standardization in the amount of tissue that was biopsied. More precisely, standard solid-liquid extraction (SLE) generally extracted larger amounts of metabolites/lipid species than SPME, as shown in the relevant ion maps complied in the **Supplementary File** (**Supplementary Figure 14**). Moreover, given the fact that SPME extracts *via* free concentration, *in vivo* applications of SPME can often have a difficult time detecting some cell-/membrane-/protein-bound analytes, whereas such analytes are often visible in SLE extractions from biopsies. Furthermore, a larger fraction of highly polar metabolites retrieved from the SLE extracts was specifically associated with the chemistry of the SPME extraction phase. Of note, more diversity of lipid classes/subclasses including low abundant lipid mediators were observed with the application of the SPME method likely being a result of a lack of matrix effect that normally affects ionization efficiency. Overall, SPME is able to provide balanced coverage of analytes with diverse properties and is a superior alternative to traditional extraction methods due to its minimally-invasive nature, simplicity, and ability to provide information about changes in metabolome dynamics.

### Heart Function During ESHP

As shown in **Figure 3**, heart function declined progressively during the perfusion period. Stroke work and preload recruitable stroke work, measures of left ventricular work and reserve were observed to worsen significantly throughout ESHP. Similarly, the maximum (dP/dt_max_) and minimum (dP/dt_min_) rates of developed pressure also declined significantly during ESHP.

### Metabolic Origin of Cardiac Function Decline During Experimental ESHP

A PLS-DA classification model was applied to the entire dataset for non-targeted metabolomics analysis, with the results showing the clear division of samples into 5 groups with highly distinct metabolic profiles coinciding with the time course of sampling (**Figures 4A, C**). An obvious distinction between the metabolic patterns of the *in vivo* and *ex-situ* perfused samples was observed in the 2D score plots of the PLS-DA models (**Figures 4A, C**); as a result, data from this comparison (involving only *ex-situ* perfused samples) were collated separately (**Figures 4B, D**). The supervised PLS-DA models were all found to be valid with good predictive capability and a low risk of over-fitting (**Supplementary Figure 15**).

Next, the discriminant metabolites (with VIP ≥ 1.5 and high absolute pcorr values) that contributed most to the model were identified, resulting in a total subset of 38 dysregulated analytes for both acquisition modes [i.e., ESHP/ESI+ (positive) and ESHP/ESI- (negative)]. Note that trihydroxyoctadecenoic acid, ubiquinone-1, and diacylglycerol (38:7) were detected in both ion modes. A vast majority of these discriminant metabolites were up-regulated throughout ESHP, with the most pronounced increase noted early in reperfusion.

The altered analytes were used to search the KEGG or SMPDB pathway databases. The results of these searches revealed 10 significantly perturbed metabolic pathways: 1) metabolism of polyunsaturated fatty acids (PUFAs), of which five analytes (15-deoxy-d-12,14-PGJ2, resolvin D1/D2, 8-isoprostaglandin E2/8-iso-15-keto-PGF2a, 8-iso-13,14-dihydro-15-keto-PGF2, 20-hydroxy-PGF2a) were observed to be up-regulated; 2) regulation of prostaglandin synthesis, of which two trihydroxyoctadecenoic acid isomers (9,10,13-trihydroxyoctadec-11-enoic acid and 9,12,13-trihydroxy-octadec-10-enoic acid) were observed to be down-regulated; 3) ROS generation, of which three metabolites (ubiquinone-1, cyclic 3-hydroxymelatonin, 8-Oxo-dGMP) were found to be largely up-regulated; 4) purine metabolism, with most metabolites down-regulated (hypoxanthine↑, inosine↓, adenosine↓, 5-methyldeoxycytidine↓); 5) metabolism of fatty acids (FAs), with six metabolites altered (butyrylcarnitine↓, octanoylcarnitine↓, stearoylcarnitine↑, 6-keto-decanoylcarnitine↑, dodecanedioic acid↑, 3-hydroxy-tetradecanedioic acid↑); 6) Krebs cycle, with two down-regulated metabolites (S-cysteinosuccinic acid, β-citryl-L-glutamic acid); 7) retinol metabolism, of which two metabolites (retinoyl β-glucuronide, 4-oxoretinoic acid) were seen to be up-regulated; 8) histidine metabolism, with two down-regulated metabolites (histidine, 1-methylhistamine); 9) corticosteroid metabolism; and 10) numerous lipids engaged in glycerophospholipid metabolism, cellular signaling, or turnover of pool of neutral lipids (**Supplementary Table 1** and **Figures 4E, F**).

Similarly, chemometric analysis of the perfusate samples showed clear separations among the groups in both ESI+ and ESI− modes (**Figure 5**). Metabolic features with VIP scores ≥ 1.5 in the PLS-DA analyses were extracted and retained as significant for downstream analysis. The quality of the generated PLS-DA models was evaluated *via* internal cross-validation. High R^2^X and Q^2^ values were observed in both the ESI+ and ESI- analyses, which confirms the presence of significant and valid global metabolic differences in the perfusate metabolome based on the time the samples were collected (**Supplementary Figure 16**).

Most alterations identified in the perfusate metabolome mimicked the pattern observed in the myocardium. However, many dysregulated analytes were only found in the former, which reflects the unique metabolome coverage of priming fluid (**Supplementary Table 2** and **Figures 5E, F**). For example, a down-regulated pathway of NO generation was only observed in the perfusate analysis, wherein (a)symmetric dimethylarginine and isoxanthopterin were detected. Other altered pathways included linoleic acid metabolism and mitochondrial phospholipidome remodeling. Together, these results show that the dysregulated pathways identified in each metabolomic/profiling pipeline complement one another, and that the use of such an integrated approach can provide an unprecedented view of pathway deregulation (**Supplementary Tables 1, 2**).

### Metabolomic Phenotype of Clinical ESHP

As a proof-of-concept, 2 human hearts underwent ESHP with the aim of validating the experimental protocol and the metabolomic findings (**Figures 6**, **7**). First, principal component analysis (PCA) was performed to enable visual inspection of clustering patterns. **Figure 6** shows a graphical representation of the PCA scores for both cases and two ionization modes, along with the obvious trends for the separation of all clusters (describing a particular state/time interval of heart perfusion) in a metabolic dataset. Furthermore, to identify the metabolite changes contributing most to the distinction between study groups, the variables exerting the greatest influence on the structure of the loading plot data were extracted. The alterations in the metabolomic pattern throughout ESHP largely converged with those observed in the porcine ESHP model, pointing to amplified inflammatory response, oxidative stress, dysregulated purine and FA metabolism, and perturbed cellular lipid signaling. Notably, alterations in cardiolipin synthesis and mitochondrial membrane remodeling were unique to the human heart samples (**Supplementary Table 3**). Nevertheless, these results confirmed the performance of the proposed assay with respect to the comprehensive profiling of heart-specific metabolites.

As in the pre-clinical setting, perfusate analysis was conducted; the results of this analysis are shown in **Figure 7** and **Supplementary Figures 17, 18**. Relevant PCA score plots revealed major grouping patterns among the samples, as well as differences in the metabolite distribution pattern responses at different time intervals. However, the causative factors responsible for the different clustering on the score plots were not evaluated, as our primary goal was to validate the feasibility of the analytical approach; hence, a greater number of case cohorts would be required to draw reliable biological conclusions.

## Discussion

*Ex situ* perfusion of the heart has demonstrated its capacity to increase the donor heart pool by successfully allowing the transplantation of ECD (34) and DCD (10) hearts. Furthermore, ESHP has shown encouraging results, with comparable outcomes to standard NDD transplants (10). Concomitant with the use of ESHP is a progressive decline in myocardial function during ESHP. By restoring oxygenation and providing metabolic substrates, machine perfusion potentially allows for the correction of metabolic derangements caused by IRI (9). The ability to adequately resuscitate and preserve donor hearts in order to expand the donor pool will be enhanced by expanding our knowledge of the metabolic deficiencies specific to these organs. Unfortunately, the optimization of current perfusion protocols is limited by a lack of understanding regarding these metabolic changes. Therefore, the data presented herein has the potential not only to improve pretransplant assessments of donor hearts, but also to inform therapies aimed at treating dysfunctional organs, as seen in other solid-organ machine-perfusion studies (13, 14).

Our goal was to characterize the metabolic changes that occur with the associated myocardial functional decline during ESHP. Our state-of-the-art metabolomics platform allowed us to identify a large number of significantly altered metabolites, with comparable results to conventional biopsies. Overall, our data highlighted the dynamic nature of the metabolic alterations that occurred during perfusion.

Specifically, our results indicated that uncontrolled inflammation was the most pronounced feature of the affected cardiac metabolome and an anticipated driver of the formation of enhanced interstitial edema. Although the acute inflammatory response triggered by exposure to the extracorporeal circuit is a well-recognized process that is considered analogous to the response associated with cardiopulmonary bypass (11, 35), putative causative factors and changes in acute inflammation over time have yet to be studied. In this work, we demonstrated that the first hours of reperfusion are associated with a strong release of arachidonic acid (AA) metabolites being a result of its autoxidation or COX-based conversion (36). In turn, these potent products, such as 8-isoprostaglandin E2, 8-iso-15-keto-PGF2a, 20-hydroxy-PGF2a, and thromboxane B2, act as proinflammatory signals that play a role in chemotaxis, the migration of inflammatory cells, leukocyte vascular adherence, and increased vascular permeability (36). The release of proinflammatory lipids was evident during the first 1.5 hours of perfusion and again after 4 h of reperfusion; however, further studies are warranted to indicate causative factors of amplified inflammatory response in the late period of the perfusion (accelerated hemolysis, leukocyte damage or depletion of tissue and perfusate antioxidants, to name a few). A significant increase in the anti-inflammatory mediator, 15-deoxy-d-12,14-PGJ2 (37), was also evident during the early reperfusion period (1.5 h). While it is currently accepted that the acute inflammatory process may be protective, persistent inflammation may result in significant injury if not counterbalanced by resolution and temporal lipid mediator class switching (38, 39). In this context, actively resolving inflammation involves not only the effective removal of proinflammatory stimuli, but also the biosynthesis of specialized pro-resolving mediators. Among PUFA metabolites, the n-3 series possesses the capacity to control the resolution of inflammation by inducing the synthesis of D and E series resolvins with potent anti-inflammatory and immunomodulatory activities (39, 40). Our data confirms the rapid generation of D-series resolvins and suggests that, similar to AA, docosahexaenoic acid (DHA) may undergo enzymatic oxidation at the onset of the reperfusion period. Altogether, depending on their metabolic phenotype and the timing of the release of PUFA-derived lipid mediators, PUFAs can either foster proinflammatory stimuli or force the inflammatory response towards a resolution phase to enable tissues to restore their function. Thus, the consequences of an inflammatory event are highly dependent on which biological pathways are favored.

Of interest is the observation that endothelial cell injury occurs much earlier and more severely than cardiomyocyte injury upon reperfusion, and potentially involves mitochondria (41). Although mitochondrial reactive oxygen species (ROS) can serve as secondary messengers and are essential in cell signaling, their presence at high levels induces oxidative stress, cellular senescence, and apoptosis (42). We observed an enhanced oxidative stress response alongside increased levels of mitochondrial markers of excessive ROS production (ubiquinone-1, cyclic 3-hydroxymelatonin, 8-oxo-dGMP, L-cystine, and methionine sulfoxide). Given that endothelial cells have relatively low mitochondrial content compared to cardiomyocytes, the mitochondria in these cells play a critical role in regulating signaling responses rather than in producing energy (41). Specifically, these responses may orchestrate intracellular processes such as cell proliferation/migration, angiogenesis, metabolism, mitochondrial permeability transition, cellular adhesion molecule expression, regulation of microvascular permeability, and anticoagulation. Furthermore, our data indicated impaired NO generation as manifested by the accumulation of a NOS inhibitor (dimethylarginine) and a metabolic breakdown product of BH_4_ (isoxanthopterin), which have both been recognized as contributors to endothelial dysfunction (43). Thus, chronic inflammation, accelerated oxidative stress, and reduced NO bioavailability result in compromised endothelial function and may be prominent features of profoundly impaired vessel function during prolonged ESHP (21). This may have direct impact on myocardial recovery, as endothelial injury could be the main cause of the “no-reflow” phenomenon that sometimes follows blood reperfusion and leads to the development of cardiac allograft vasculopathy (44).

Glucose is the primary energy substrate in ESHP perfusates (3, 11, 35). Our data indicates that glucose is not utilized optimally in this setting, which is evidenced by the disruption of mitochondrial bioenergetics, impaired β-oxidation, and the cardiac accumulation of harmful lipid species. The accumulation of long-chain acylcarnitines indicates incomplete mitochondrial FA oxidation, whereas a massive buildup of dicarboxylic acids suggests the activation of an alternative pathway of FA degradation: ω-oxidation. Several studies have demonstrated that, when β-oxidation is impaired, ω-oxidation can prevent excessive accumulation of long-chain FAs, and therefore the adverse effects related to the accumulation of these FAs in pathological conditions (45). These results also indicate that optimizing metabolic substrate utilization during ESHP can dramatically improve donor heart preservation. However, under normal conditions where the oxidative metabolism of glucose and FAs and their contribution to ATP production is tightly regulated (46), reliance on one type of energy substrate is not ideal. As shown in this work, altered physiological states such as ischemia/reperfusion injury and *ex situ* preservations modify normal metabolic pathways and normal substrate utilization by stimulating or inhibiting oxidation.

Impaired FA metabolism can lead to cardiac lipotoxicity, which refers to the accumulation of intracellular lipid species and the development of myocardial dysfunction (47). This condition is primarily driven by diacylglycerols (DAGs) and ceramides, which also increased upon reperfusion in our study. This reaction suggests that reducing the accumulation of toxic lipids by stimulating FAO could improve the preservation of myocardial function during ESHP, and that switching to glucose long-term may be maladaptive in nature.

Purine metabolism was also affected by ESHP, which was evidenced by the rapid depletion of adenosine and its downstream metabolic by-product, inosine, and an accumulation of hypoxanthine. This result is in line with previous findings indicating that adenine nucleotide catabolism during ischemic events results in the intracellular accumulation of hypoxanthine (48). The moderate increase in hypoxanthine levels in cardiac tissue observed in this study may suggest moderate ischemia following reperfusion.

Furthermore, our findings demonstrated the presence of adrenocorticoids, such as corticosterone and aldosterone, in *ex situ* preserved hearts. There is increasing evidence that gluco- and mineralocorticoids may accumulate in the cardiac interstitium and modulate cardiovascular homeostasis *via* many downstream effects (49). While we observed the dysregulation of such a steroidogenic system in the *ex situ* perfused heart, further results are needed to define its overall physiological relevance.

In addition, alterations in the lipidome were also identified. A progressive depletion in docosapentaenoic acid (DPA) and docosahexaenoic acid (DHA), downstream products of linolenic acid catabolism, was observed, suggesting a reduction of their availability. We believe this result is associated with mitochondrial dysfunction, as cardiac mitochondrial phospholipid acyl chains modulate respiratory enzymatic activity, and extensive rearrangement of the cardiac phospholipidome has also been shown in several disease states (50). Although it remains unclear how this remodeling promotes mitochondrial dysfunction, several studies have suggested that linoleic and docosahexaenoic acid exert a pivotal role and progress towards pathology when the balance is diverted to the rapid incorporation of DHA into the mitochondrial membranes (50, 51). Notably, our results also provided evidence that the increased availability of some phosphatidylserine (PS) and phosphatidylethanolamine (PE) species may be linked to membrane lipid remodeling and the loss of membrane phospholipid asymmetry. These results corroborate previous findings that suggest the involvement of both phospholipids in crucial biological processes such as apoptosis and cell signaling, and that their translocation from the inner leaflet to the outer leaflet of the membrane precedes the apoptotic cell death associated with the reperfusion following an ischemic insult (52). However, further investigation is required to verify the potential connection between PE/PS species and accelerated apoptosis in our experimental setting.

Adding to the above, to the best of the authors’ knowledge this is the first study on evaluation of altered global cardiac metabolome during ESHP. Previous studies were focused on a narrow scope of changes, namely on alterations in energy substrate utilization, accelerated inflammatory response and oxidative stress or ultimately the loss of coronary artery regulation which might be directly associated with endothelial dysfunction; nonetheless, they present a valuable insight in the context of metabolic alterations discussed in the current study (35, 53–55). Precisely, Hatami S. et al. have proven that functional decline during ESHP has a metabolic basis and therefore might be reversible (53). During extended 12 hours porcine hearts perfusion they observed decoupling of glycolysis and the TCA cycle at the level of pyruvate kinase and revealed that strategy with simultaneous infusion of insulin and glucose appeared insufficient for maintenance of metabolic integrity during ESHP. This is in line with our observations that have shown that adverse changes in energy metabolism/energy substrate utilization might represent causative factors contributing to deteriorating contractile function. In another study, Qi X et al. demonstrated that regulation of coronary artery function was disturbed during 12 hours normothermic perfusion leading to excessive coronary blood flow over time (54). Whether the loss of coronary artery regulation causes the decline of cardiac function requires further investigations; however, it is expected that this phenomenon may be related to endothelial damage which we also showed in our studies. Further, accelerated immunity and oxidative stress responses were identified in a rodent as well as porcine model of ESHP. In the study conducted by Hatami S et al. both inflammation and endoplasmic reticulum stress responses were significantly induced during porcine ESHP presumably contributing to the deteriorating heart function yet representing a potential therapeutic target to improve the donor heart preservation (35). In turn, Li J et al. demonstrated that DCD, and non-DCD rat hearts presented similarly in apoptosis, oxidative stress, inflammatory response, and myocardial infarction changes though the DCD hearts had lower energy storage when compared against non-DCD hearts (55). Our study corroborates the above research findings underpinning activation of innate immune, and oxidative stress responses as playing a critical role in the functional decline of the *ex situ* preserved heart.

Finally, our study has several important limitations; for example, the use of a relatively small sample size. Nevertheless, our findings for the porcine model demonstrated significant metabolic changes throughout ESHP, which corresponded to findings using human hearts. Furthermore, the use of multiple comparison corrections and false-discovery rate control—which was employed in the untargeted metabolomic and lipidomic profiling to avoid false discoveries—can compensate for the small sample size. Adding to the above, given that the left ventricle is thicker and more muscular than the right one, and generally subjected to a greater load, this may also affect metabolomics pattern thus in future studies a clear separation of changes identified in each of the chambers with a more standardized approach to sampling at a nearby location (over time) was planned. Apart from that, among the most altered metabolic pathways/biochemical processes during prolonged ESHP we identified amplified inflammatory and oxidative stress response, endothelial injury, compromised energy substrate utilization, the disruption of mitochondrial bioenergetics, and the accumulation of harmful lipid species. Of note, all of the above-mentioned alterations were identified both in the healthy juvenile pig hearts and the declined human donor hearts characterized by various metabolic baseline. The induction of inflammatory responses and oxidative stress, endothelial injury, next to not efficient energetic fuel utilization, and accumulation of harmful metabolic intermediates might be related with the nature of applied extracorporeal circulation and sub-optimal composition of the perfusate; thus warrants further optimization of the perfusion protocol. The myocardial tissue challenged and stressed with these metabolic alterations, may be a source of uncontrolled release of diverse lipid mediators, cellular signaling molecules, metabolic by-products that ultimately mask graft conditions qualifying or disqualifying its for transplant. Furthermore, the identification of the early markers for the quality of donor hearts was beyond the scope of this article, but studies using the proposed approach are in progress, with a greater number of cases and to subsequently demonstrate clinically relevant observations and point to biomarkers that may facilitate in these applications. Adding to the above, first interventional studies aiming to remove metabolic waste products which accumulation was apparently seen in the current study are underway with encouraging results. Ultimately, the use of human hearts (as in this study) is prone to significant heterogeneity, as there is significant variance in not only the demographic data, but also in metabolic variability based on medical history and ischemic times. However, it was not authors’ intention to build on them strong biological conclusions rather to indicate trends that appeared to coincide with these identified in the pre-clinical setting. Regardless, further studies involving more numerous cohorts of cases are underway to investigate in-depth changes in the metabolism of *ex vivo* preserved human hearts. In addition, the authors are aware that different ischemic time between various experimental settings of organ procurement and perfusion presented in this study may directly affect the severity of ischemia-reperfusion injury and inflammation/oxidative stress. Though ischemic time was standardized to 1 hour in our porcine model, we were unable to standardize the ischemic time for the 2 human hearts used but this was only meant as a pilot study to assess if our sampling approach/metabolomic protocol and results would be in line with our translational model.

In conclusion, in this paper, we documented a state-of-the-art analytical pipeline for the comprehensive metabolic profiling of *ex situ* preserved hearts. We coupled an SPME chemical biopsy tool and high-resolution mass spectrometry, which enabled the detection of a broad range of bioactive metabolites. Several unique features of this new sampling approach were presented, namely: 1) its minimal invasiveness and negligible disruption of the system being investigated; 2) its ability to allow repeated extractions, thereby permitting studies of processes at different times or localizations within the same system; 3) the feasibility of evaluating regional changes in metabolite patterns; 4) its ability to capture the elusive (non-stable) fraction of metabolites; and 5) its potential for rapid diagnostics/treatment tailoring by directly coupling SPME probes with highly specific mass detectors. This is the first study to examine the relationship between the dysregulation of the cardiac metabolome and declining myocardial function during ESHP. Several altered metabolic pathways were demonstrated, with emphasis on increased inflammatory and oxidative stress response and compromised substrate utilization. Targeting these features may improve the recovery and preservation of cardiac functionality during ESHP. Finally, the combination of perfusion parameters and metabolomics can uncover various mechanisms of organ injury and recovery, which can potentially help distinguish organs that are transplantable from those that should be discarded. Adding to the above, owing to SPME’s simplicity, flexibility of design and versatility, it emerges as a promising technology, with a myriad of possible future applications going beyond organ status assessment and directed at providing new insights into the processes occurring in the biological system investigated. The proposed chemical biopsy tool could potentially be used as a medical device due to its minimally invasive nature and operational parameters that are compatible with surgical procedures. It is anticipated that SPME-based devices will find application especially in oncology (including neurooncology) offering efficient extraction and preservation of biological material. Apart from that, implementation of SPME in combination with rapidly developing technologies, including portable reading devices using ion mobility devices and optical spectroscopic techniques, such as fluorescence or Raman imaging may create a precision medicine tool at the bedside. Once the sensitive markers to monitor are selected, it is possible to collect meaningful information in less than an hour (considering the time needed for material collection/extraction) thus opening new avenues for accurate, rapid diagnostics.

## Data Availability Statement

The raw data supporting the conclusions of this article will be made available by the authors, without undue reservation.

## Ethics Statement

The studies involving human participants were reviewed and approved by Province of Ontario’s organ procurement organization. The patients/participants provided their written informed consent to participate in this study. The animal study was reviewed and approved by Institutional Review Board (IRB) at the University Health Network (UHN; Toronto, ON, Canada) and University of Waterloo’s Research Ethics Board.

## Author Contributions

Project conceptualization, funding, and supervision: JP, MB, and FB; experimental design: MO, RVPR, and RS; devices manufacturing and initial experimental trials: MO, RVPR, FY, LX, MA; participation in performing the experiments, data acquisition and interpretation: MO, RVPR, FY, JA, MY, RR, MA, VB; manuscript writing: MO, RVPR.; all involved authors revised and accepted the submitted version of the manuscript.

## Funding

This work was supported by the Natural Sciences and Engineering Research Council (NSERC) of Canada through the Industrial Research Chair (IRC) program [#IRCPJ 184412-15].

## Conflict of Interest

The authors declare that the research was conducted in the absence of any commercial or financial relationships that could be construed as a potential conflict of interest.

## Publisher’s Note

All claims expressed in this article are solely those of the authors and do not necessarily represent those of their affiliated organizations, or those of the publisher, the editors and the reviewers. Any product that may be evaluated in this article, or claim that may be made by its manufacturer, is not guaranteed or endorsed by the publisher.
