# Supplementary material for: Dynamic Metabolic Changes During Prolonged Ex Situ Heart Perfusion Are Associated With Myocardial Functional Decline
Source: Front Immunol. 2022 Jun 24;13:859506. doi: 10.3389/fimmu.2022.859506 (PMC9267769; doi:10.3389/fimmu.2022.859506)
Supplement: Supplementary file 1 [file DataSheet_1.docx]

Supplementary Information

**Dynamic Metabolic Changes During Prolonged Ex Situ Heart Perfusion Are Associated with Myocardial Functional Decline**

Mariola Olkowicz^1,2^ †, Roberto Vanin Pinto Ribeiro^3, 4^ †, Frank Yu^3^, Juglans Souto Alvarez^3^, Liming Xin^3^, Miao Yu^5^, Roizar Rosales^3^, Mitchell Brady Adamson^3^, Ved Bissoondath^3^, Ryszard T. Smolenski^6^, Filio Billia^7,8^, Mitesh Vallabh Badiwala^3,4,8#^, Janusz Pawliszyn^1 #^*

^1^ Department of Chemistry, University of Waterloo, Waterloo, ON, Canada

^2^ Jagiellonian Centre for Experimental Therapeutics (JCET), Jagiellonian University, Krakow, Poland

^3^ Division of Cardiovascular Surgery, Peter Munk Cardiac Center, Toronto General Hospital, University Health Network, Toronto, ON, Canada

^4^ Division of Cardiac Surgery, Department of Surgery, Faculty of Medicine, University of Toronto, Toronto, ON, Canada

^5^ Department of Environmental Medicine and Public Health, Icahn School of Medicine at Mount Sinai, New York, USA

^6^ Department of Biochemistry, Medical University of Gdansk, Gdansk, Poland

^7^ Toronto General Hospital Research Institute (TGHRI), University Health Network, ON, Canada

^8^ Ted Roger’s Center for Heart Research, University Health Network, ON, Canada

^†^contributed equally to this work;

^#^co-senior authors;

*corresponding author: janusz@uwaterloo.ca; Tel: +1 519-888-4641; Fax: +1 519-888-4348.

Heart sampling – 1^st^ case (PFP-based mode)

**A** **B**

**ESI+**


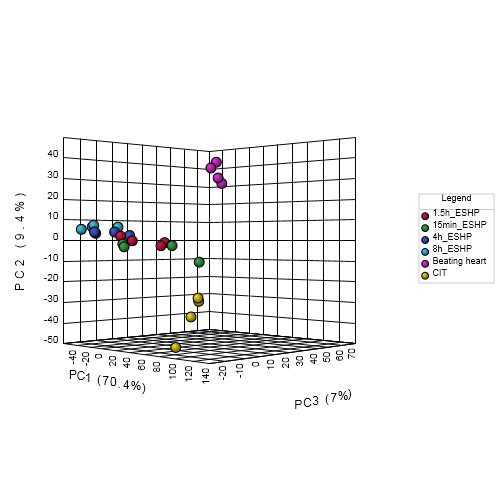

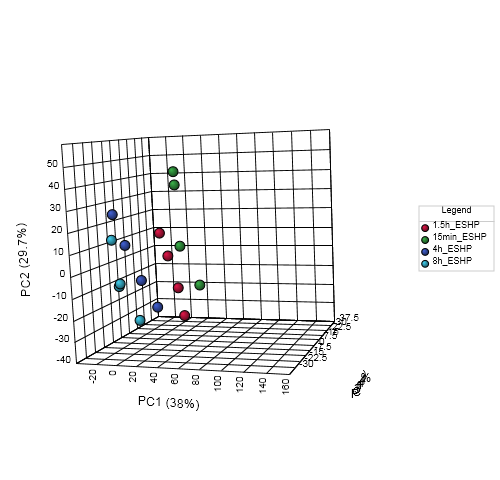


**C** **D**


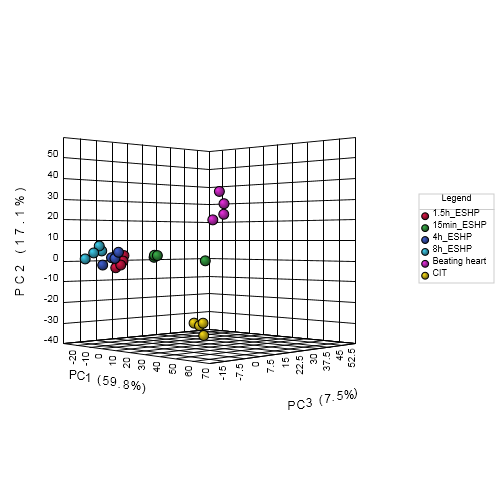

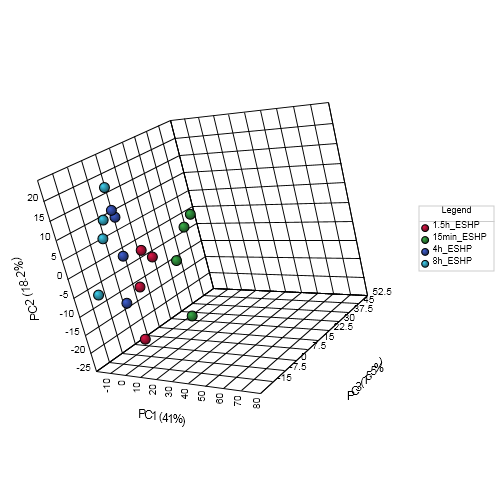


**ESI-**

**Supplementary Fig. 1.** 3-dimensional PCA score plots comparing the LC-MS metabolomic profiles of six (**A**, **C**) and four (**B**, **D**) conditions corresponding to relevant sampling time points during heart perfusion in the **1^st^ case**. The models were created for the features detected in positive (**A**, **B**) and negative (**C**, **D**) ion/**PFP-based** mode. The samples from different groups are presented using different colors (colored dots represent individual samples): **pink**—samples collected from beating heart; **yellow**—samples collected under cold ischemic time; **green**—samples collected 15 min after commencing ESHP; **red**—samples collected 1.5 h after commencing ESHP; **dark blue**—samples collected 4 h after commencing ESHP; **light blue**—samples collected 8 h after commencing ESHP. After applying the relevant processing criteria, the total number of features left for the analysis was 1333 for ESI+ mode and 668 for ESI- mode. A clear discrimination between the metabolomic patterns for samples collected from a beating heart (or under CIT) and during ESHP is observable.

Heart sampling – 2^nd^ case (PFP-based mode)

**A** **B**


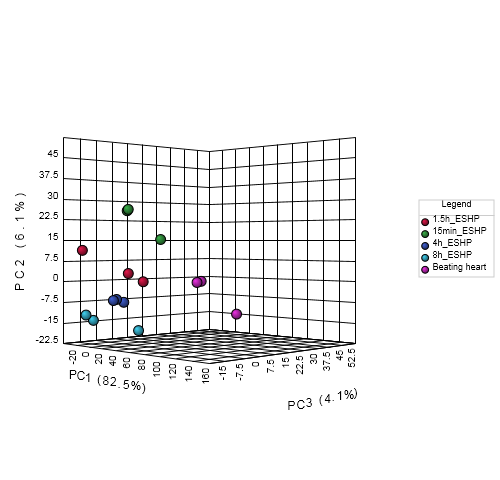

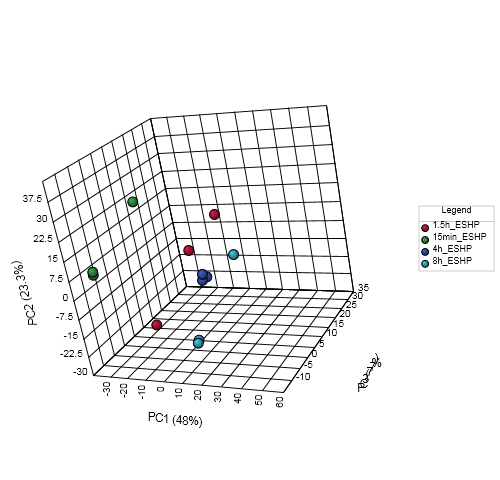


**ESI-**

**ESI+**

**C** **D**


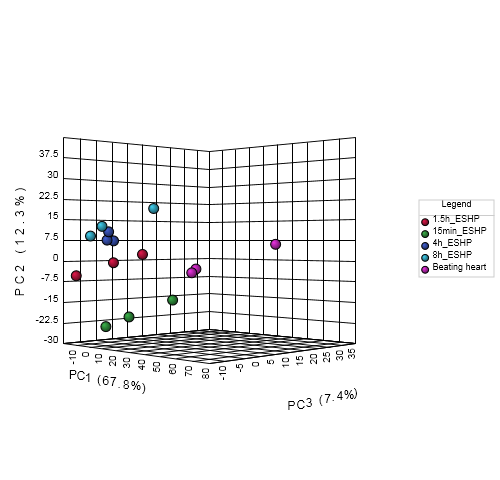

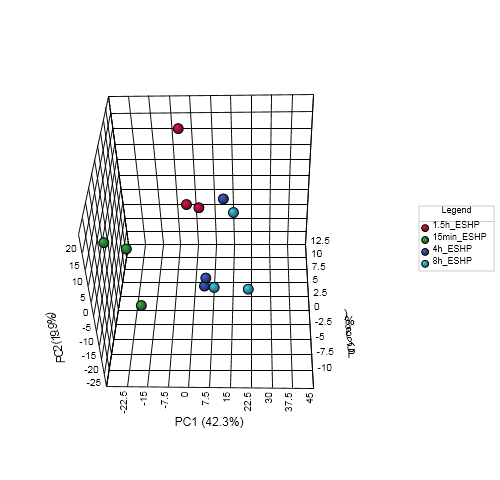


**Supplementary Fig. 2.** 3-dimensional PCA score plots comparing LC-MS metabolomic profiles among five (**A, C**) and four (**B, D**) conditions corresponding to relevant sampling time points during heart perfusion in the **2^nd^ case.** The models were created for the features detected in positive (**A**, **B**) and negative (**C**, **D**) ion/**PFP-based** mode. After applying the relevant processing criteria, the total number of features left for the analysis was 1249 for ESI+ mode and 668 for ESI- mode. Similar to the previous case, a distinct discrimination between metabolic profiles for samples collected during *in vivo* and *ex vivo* perfusion is observable.

Heart sampling – 3^rd^ case (PFP-based mode)

**ESI+**

**A** **B**


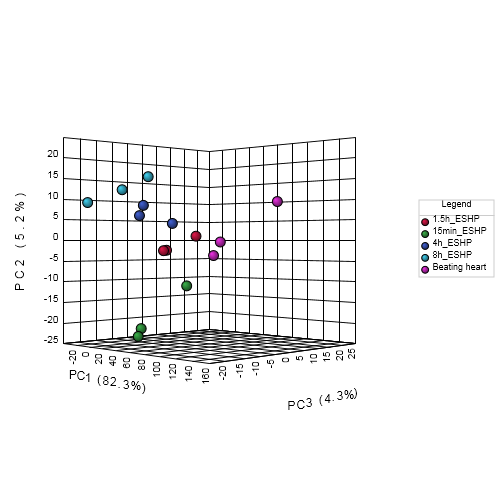

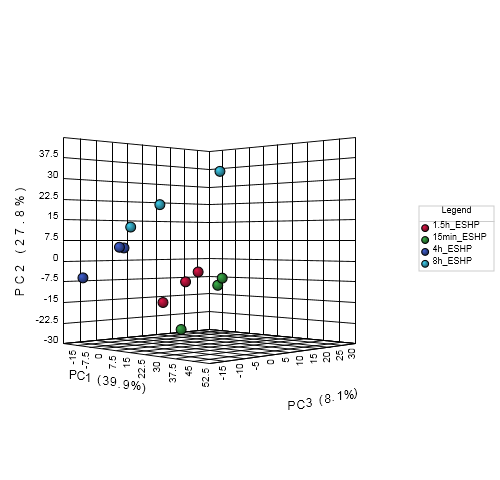


**ESI-**

**C** **D**


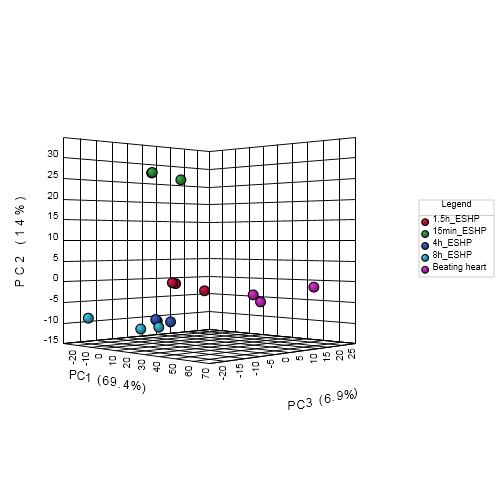

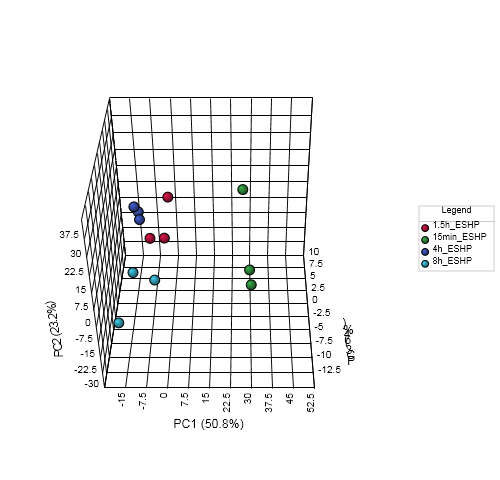


**Supplementary Fig. 3.** 3-dimensional PCA score plots comparing LC-MS metabolomic profiles among five (**A, C**) and four (**B, D**) conditions corresponding to relevant sampling time points during heart perfusion in the **3^rd^ case**. The models were created for the features detected in positive (**A**, **B**) and negative (**C**, **D**) ion/**PFP-based** mode. The total number of features left for the analysis was 1165 for ESI+ mode and 668 for ESI- mode.

Perfusate sampling – 3 cases (PFP-based mode)

**A** Arterial samples **B** Venous samples

**ESI+**


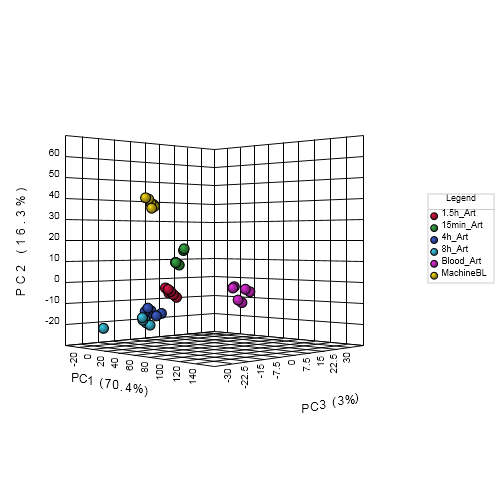

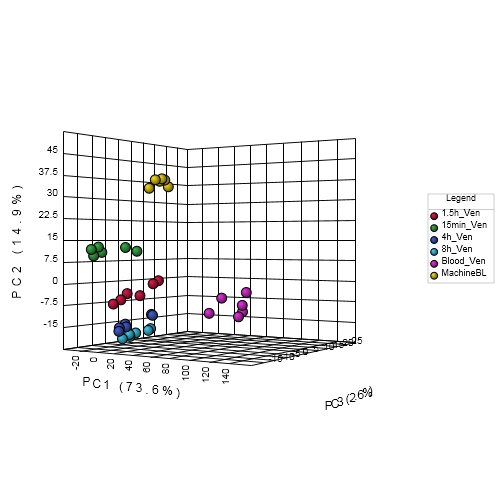


**C** **D**

**ESI-**


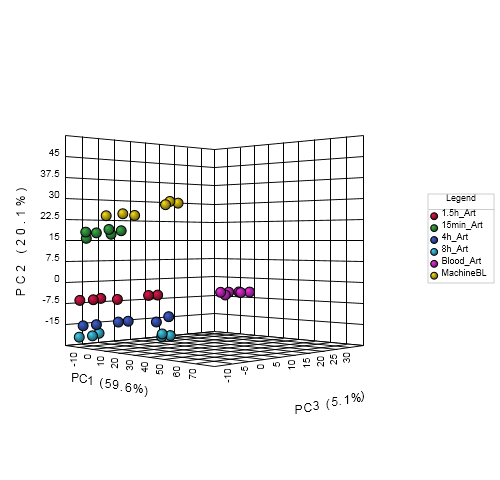

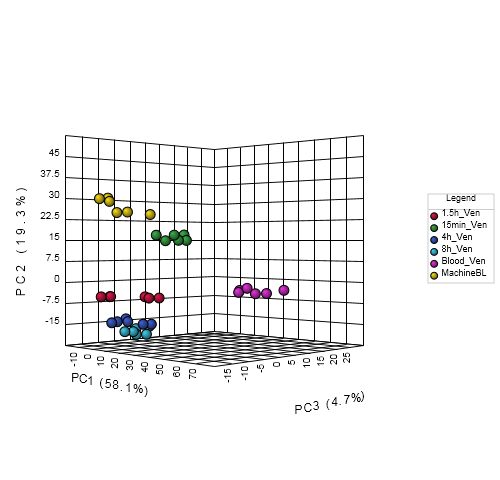


**Supplementary Fig. 4.** The principal component analysis (PCA) results for plasma/perfusate samples collected from 3 cases showed excellent discrimination of metabolic profiles based on time of sample collection when mixed-mode (MM) particles were used for analyte extraction. The models were created for the features detected in positive (**A**, **B**) and negative (**C**, **D**) ion/**PFP-based** mode. **A** and **C** depict the results for arterial (blood) sampling, while **B** and **D** depict the results for venous (blood) sampling. **Pink**—blood plasma samples; **yellow**—machine baseline samples; **green**—arterial/venous perfusate samples taken at 15 min of ESHP; **red**—arterial/venous perfusate samples taken at 1.5 h of ESHP; **dark blue**—arterial/venous perfusate samples taken at 4 h of ESHP; **light blue**—arterial/venous perfusate samples taken at 8 h of ESHP. The total number of features left for the analysis was 872 for ESI+ mode and 631 for ESI- mode.

Heart sampling – 1^st^ case (C18-based mode)

**A** **B**

**ESI+**


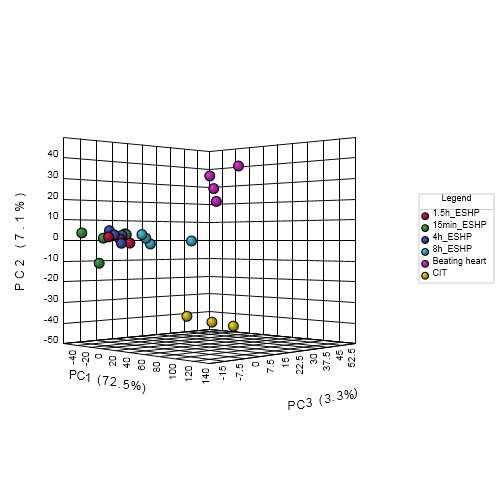

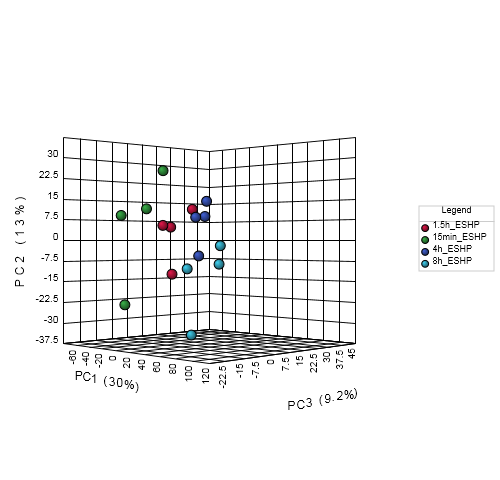


**C** **D**

**ESI-**


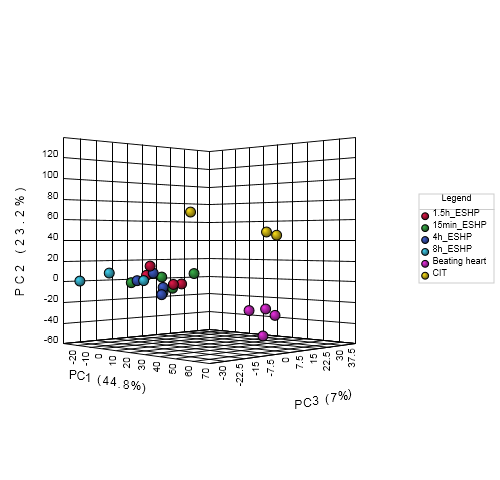

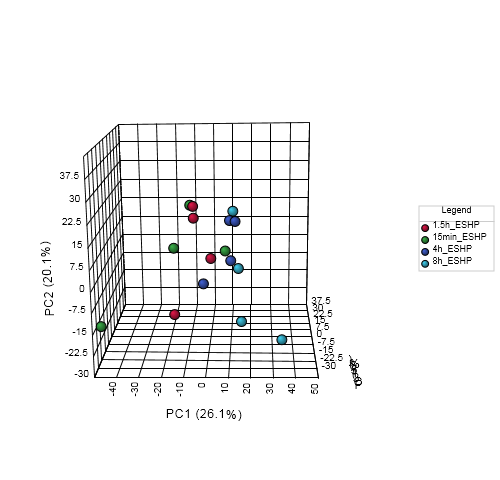


**Supplementary Fig. 5.** 3-dimensional PCA score plots comparing LC-MS metabolomic profiles of six (**A**, **C**) and four (**B**, **D**) conditions corresponding to relevant sampling time points during heart perfusion in the **1^st^ case**. The models were created for the features detected in positive (**A**, **B**) and negative (**C**, **D**) ion/**RP (reversed phase)** mode. The total number of features left for the analysis was 1874 for ESI+ mode and 1533 for ESI- mode.

Perfusate sampling – 3 cases (C18-based mode)

**A** Arterial samples **B** Venous samples

**ESI+**


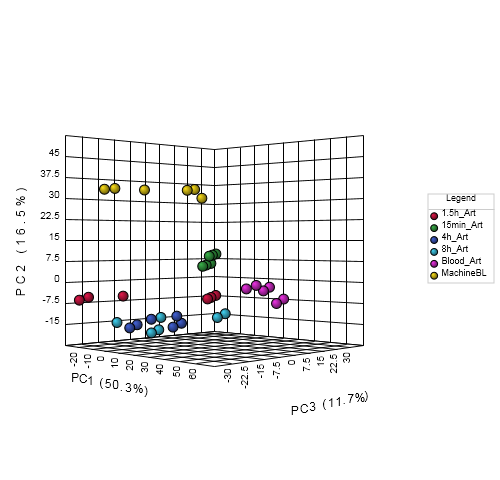

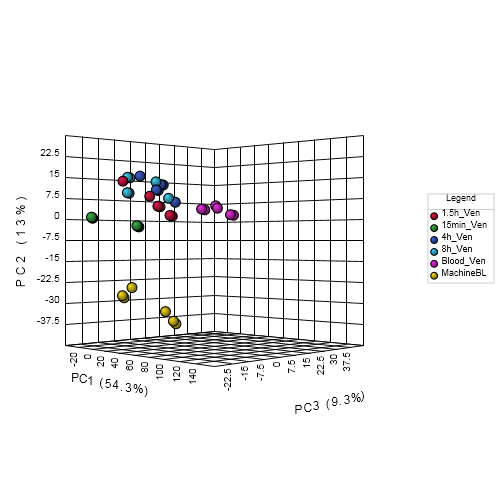


**C** **D**

**ESI-**


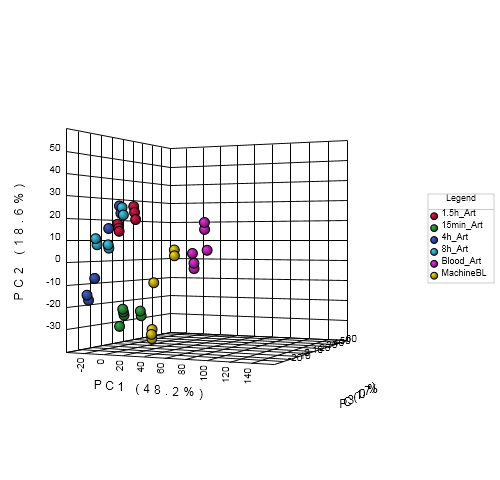

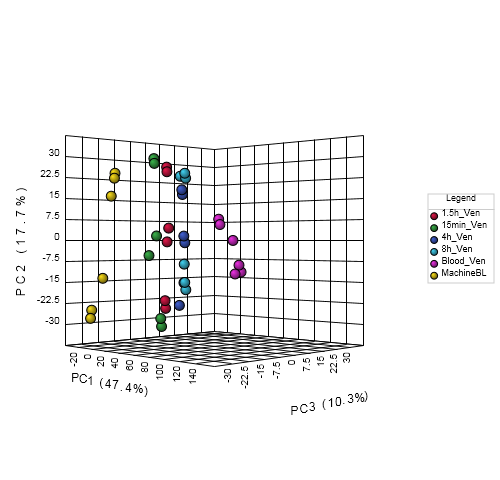


**Supplementary Fig. 6.** Principal component analysis (PCA) results for plasma/perfusate samples collected in the 3 cases when the C18 coating was used for analyte extraction. The models were created for the features detected in positive (**A**, **B**) and negative (**C**, **D**) ion/ **RP (reversed phase)** mode. **A** and **C** depict the results for arterial (blood) sampling, while **B** and **D** depict the results for venous (blood) sampling. The total number of features left for the analysis was 855 for ESI+ mode and 993 for ESI- mode. The models were confirmed to have good predictive ability; however, their predictive power was weaker compared to those built on data collected when MM fibers were used.

Heart sampling – 1^st^ case (HILIC mode)

**A** **B**

**ESI+**


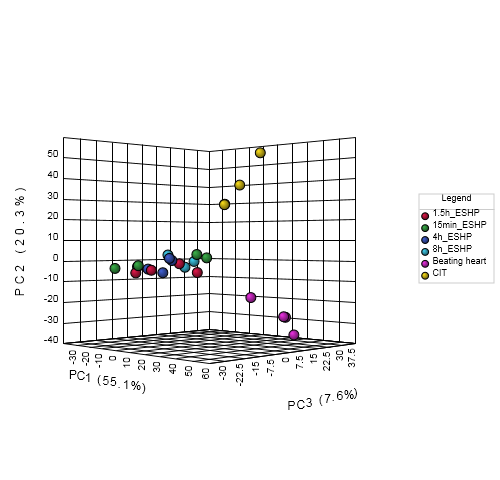

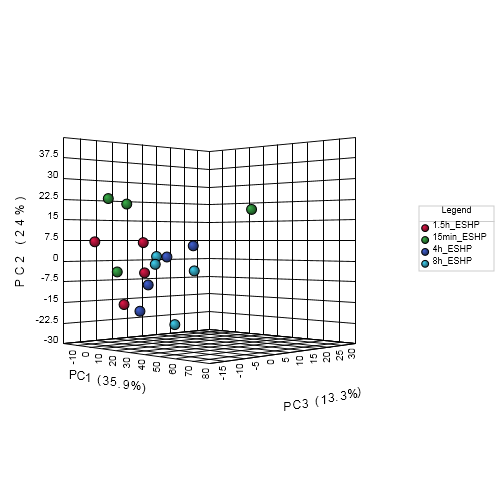


**C** **D**

**ESI-**


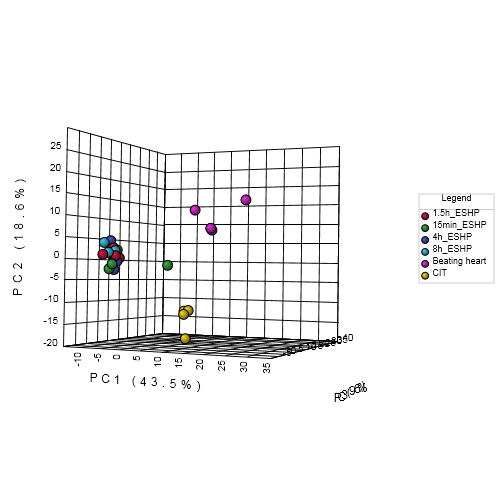

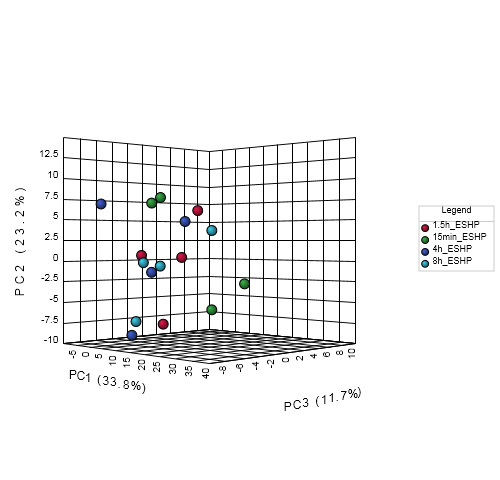


**Supplementary Fig. 7.** 3-dimensional PCA score plots comparing LC-MS metabolomic profiles among six (**A**, **C**) and four (**B**, **D**) conditions corresponding to relevant sampling time points during heart perfusion in the **1^st^ case**. The models were created for the features detected in positive (**A**, **B**) and negative (**C**, **D**) ion/**HILIC** mode. The total number of features left for the analysis was 858 for ESI+ mode and 178 for ESI- mode. Similar to the data obtained using the two previous acquisition (chromatographic) modes, a clear discrimination in metabolomic patterns could be observed among the samples collected from a beating heart (or under CIT) and during ESHP.

Heart sampling – 2^nd^ case (HILIC mode)

**A** **B**

**ESI+**


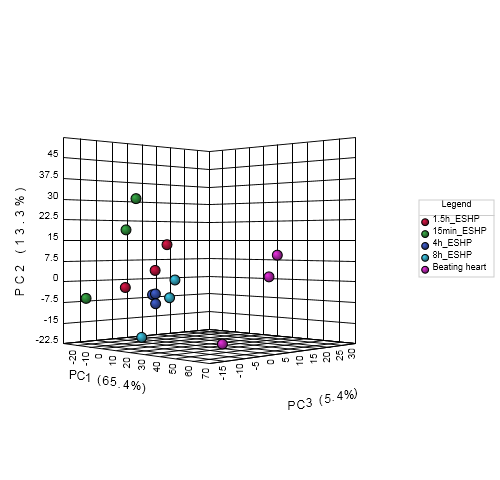

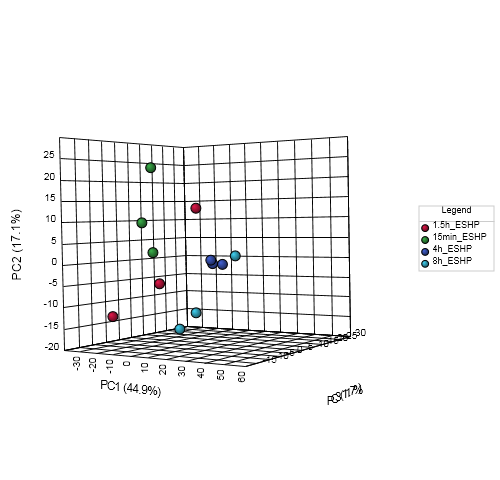


**C** **D**

**ESI-**


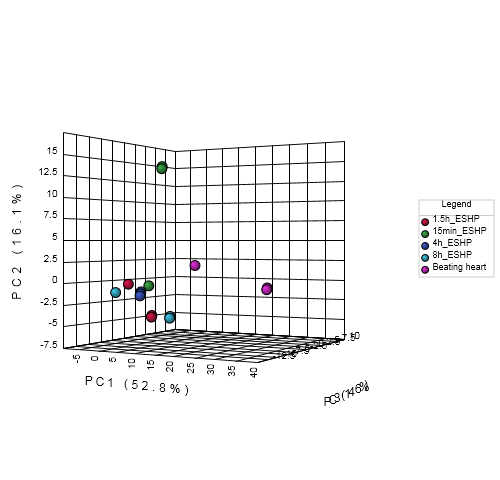

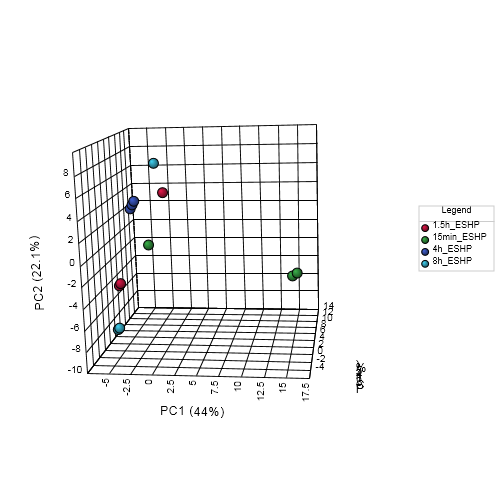


**Supplementary Fig. 8.** 3-dimensional PCA score plots comparing LC-MS metabolomic profiles among five (**A, C**) and four (**B, D**) conditions corresponding to relevant sampling time points during heart perfusion in the **2^nd^ case**. The models were created for the features detected in positive (**A**, **B**) and negative (**C**, **D**) ion/**HILIC** mode. The total number of features left for the analysis was 786 for ESI+ mode and 178 for ESI- mode. The results indicate that the models offer good predictive ability, particularly the model created in ESI+/HILIC mode. In addition, a distinct differentiation in metabolic patterns throughout ESHP is observable.

Heart sampling – 3^rd^ case (HILIC mode)

**A** **B**

**ESI+**


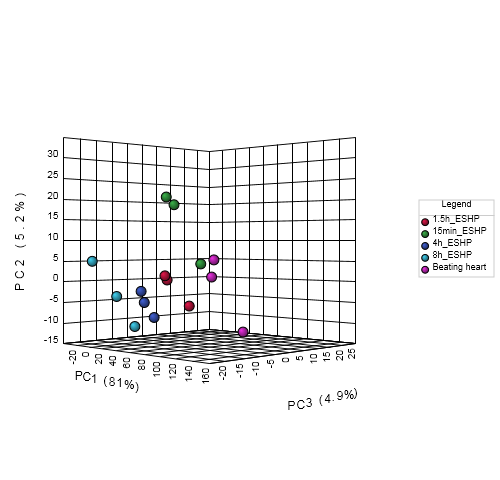

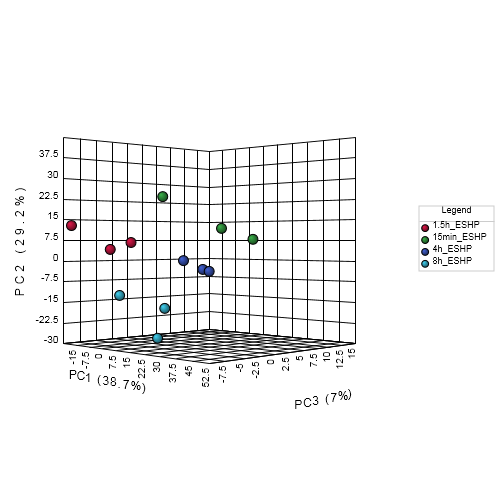


**C** **D**

**ESI-**


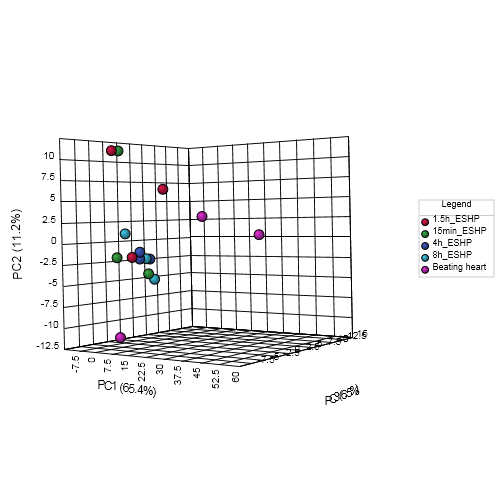

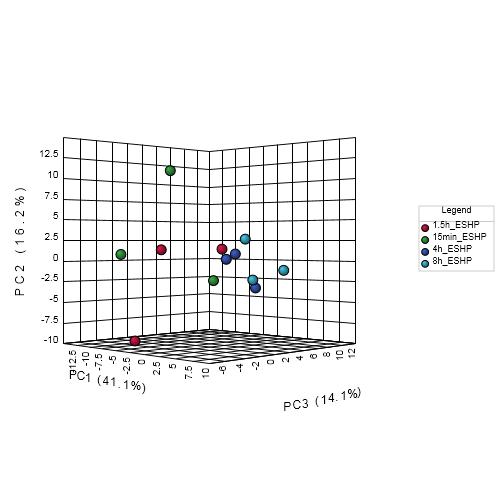


**Supplementary Fig. 9.** 3-dimensional PCA score plots comparing LC-MS metabolomic profiles among five (**A, C**) and four (**B, D**) conditions corresponding to relevant sampling time points during heart perfusion in the **3^rd^ case**. The models were created for the features detected in positive (**A**, **B**) and negative (**C**, **D**) ion/**HILIC** mode. The total number of features left for the analysis was 822 for ESI+ mode, and 178 for ESI- mode. Comparable changes in metabolomic pattern over the period of *in vivo* and *ex-situ* heart perfusion were identified in the last 2 cases studied.

Perfusate sampling – 3 cases (HILIC mode)

**ESI+**

**A** **B**


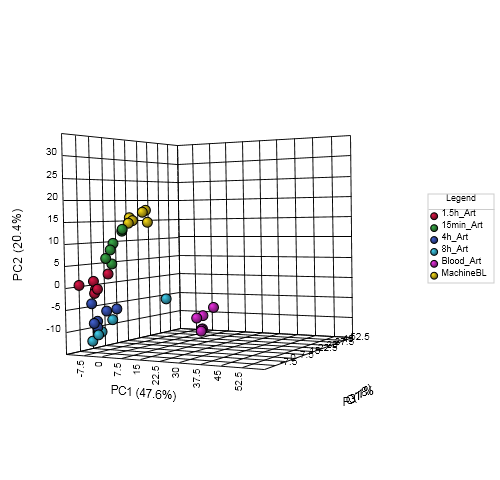

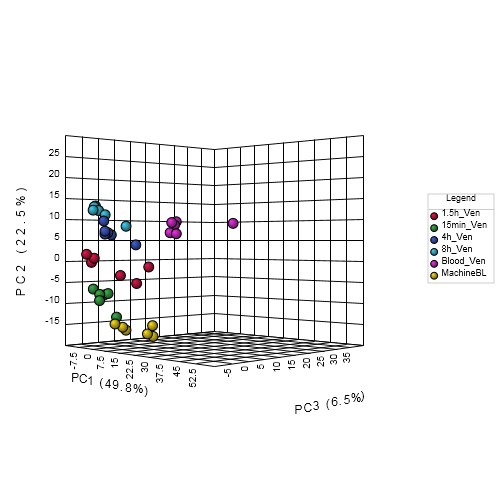


**C** **D**

**ESI-**


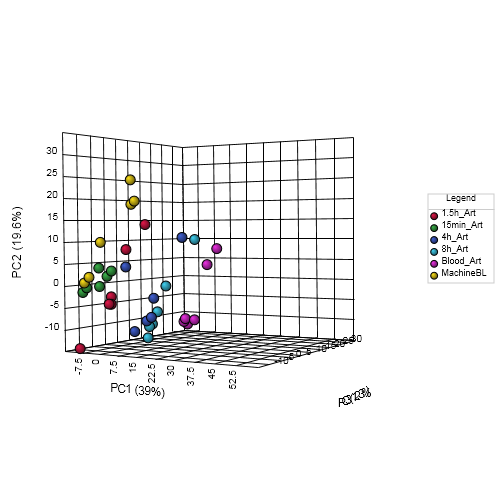

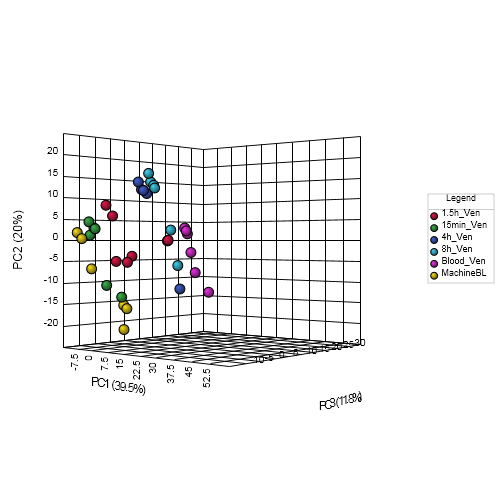


**Supplementary Fig. 10.** The principal component analysis (PCA) results for plasma/perfusate samples collected from the 3 cases showed excellent discrimination of metabolic profiles based on time of sample collection when the mixed-mode (MM) coating was used for extraction and ESI+/HILIC mode was used for analyte separation. The presented models were created for the features detected in positive (**A**, **B**) and negative (**C**, **D**) ion/**HILIC** mode. **A** and **C** depict the results for arterial (blood) sampling, while **B** and **D** depict the results for venous (blood) sampling. The total number of features left for the analysis was 214 for ESI+ mode and 180 for ESI- mode.

**PFP/ESI+**

**A**

**B**

**C**

**PFP/ESI-**

**D**

**E**

**F**

**Supplementary Fig. 11.** Base peak chromatograms for selected heart tissue (**A**, **D**) and perfusate samples (**B**, **C** and **E**, **F**) collected in PFP-based chromatographic mode.

**C18/ESI+**

**A**

**B**

**C**

**C18/ESI-**

**D**

**E**

**F**

**Supplementary Fig. 12.** Base peak chromatograms for selected heart tissue (**A**, **D**) and perfusate samples (**B**, **C** and **E**, **F**) collected in C18/ODS-based chromatographic mode.

**HILIC/ESI+**

**A**

**B**

**C**

**HILIC/ESI-**

**D**

**E**

**F**

**Supplementary Fig. 13.** Base peak chromatograms for selected heart tissue (**A**, **D**) and perfusate samples (**B**, **C** and **E**, **F**) collected in HILIC chromatographic mode.

**A**

**Supplementary Fig. 14.** Ion maps depicting feature coverage for SPME and endomyocardial biopsy (EMB) extracts collected after *in* *vivo* heart sampling, and subsequently analyzed using a reversed-phase general metabolomic method with a PFP column.

**ESI-**

**ESI+**

**B**

**A**


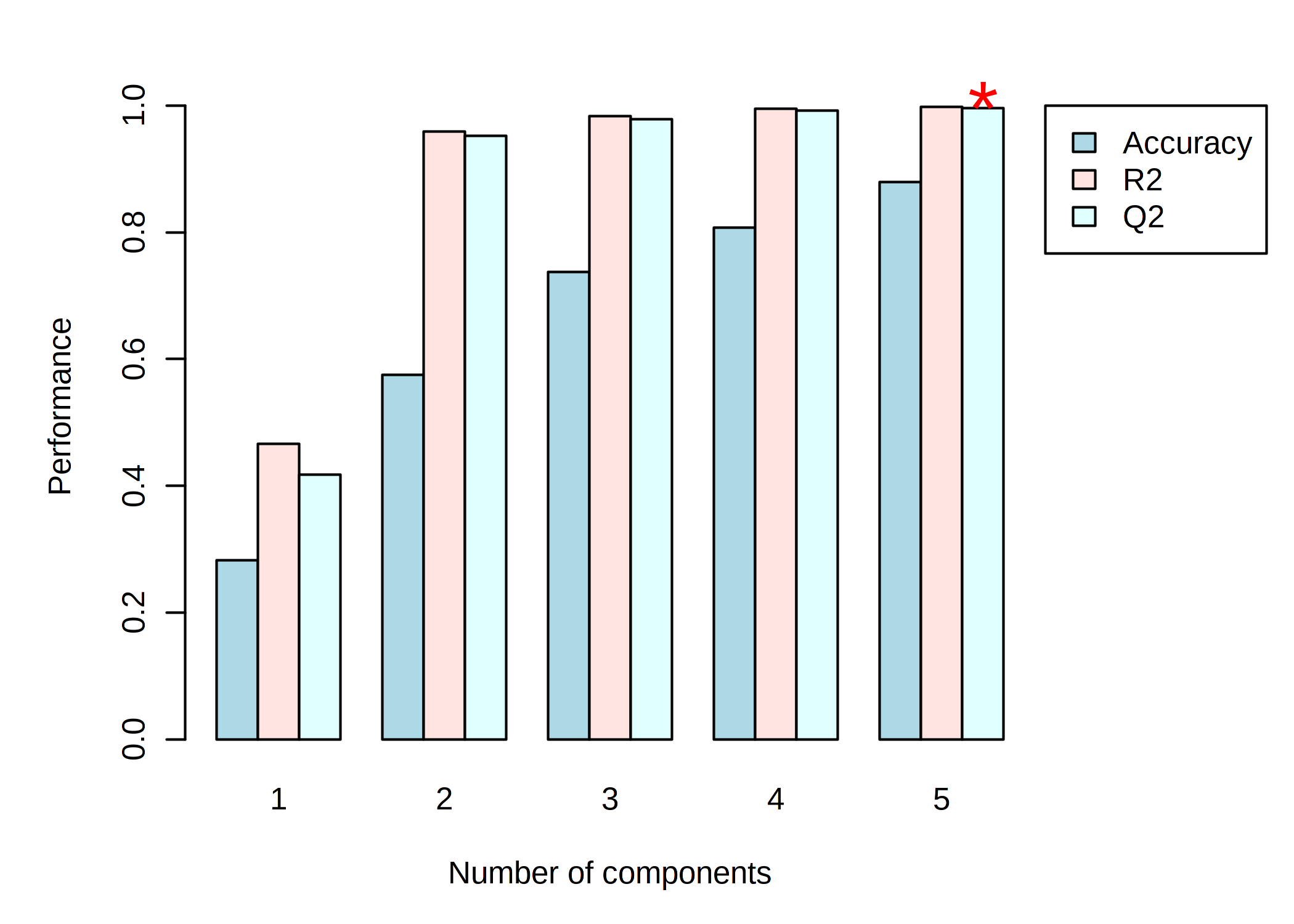

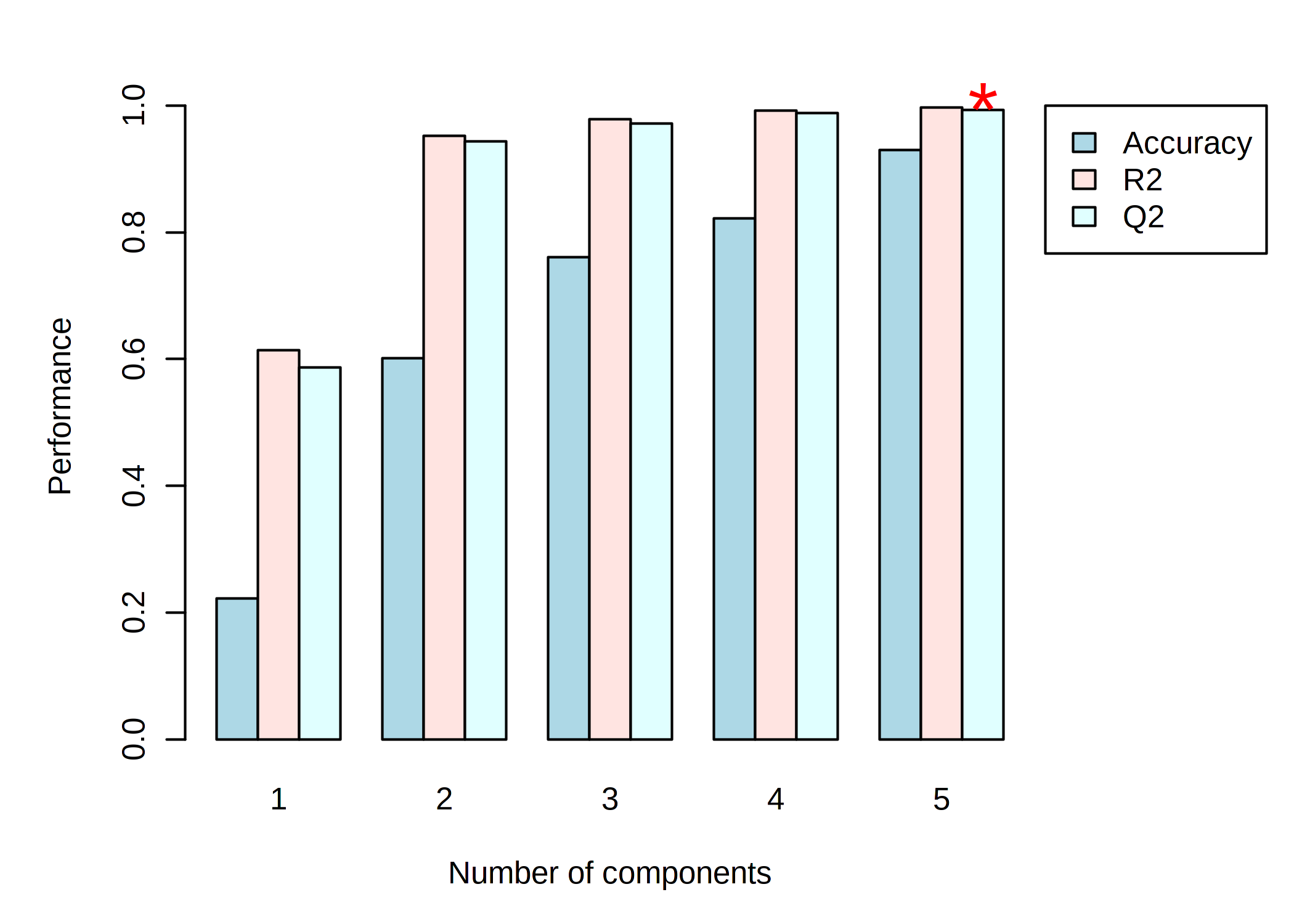


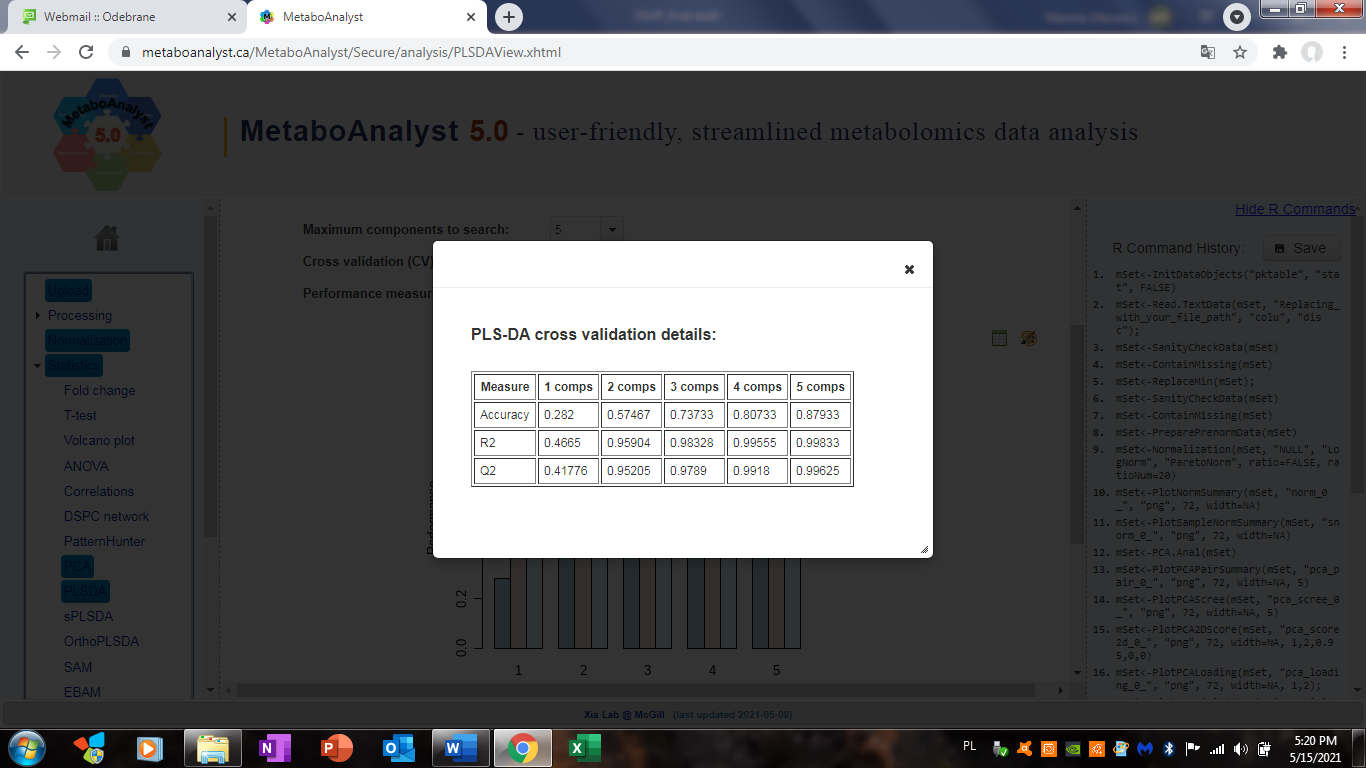

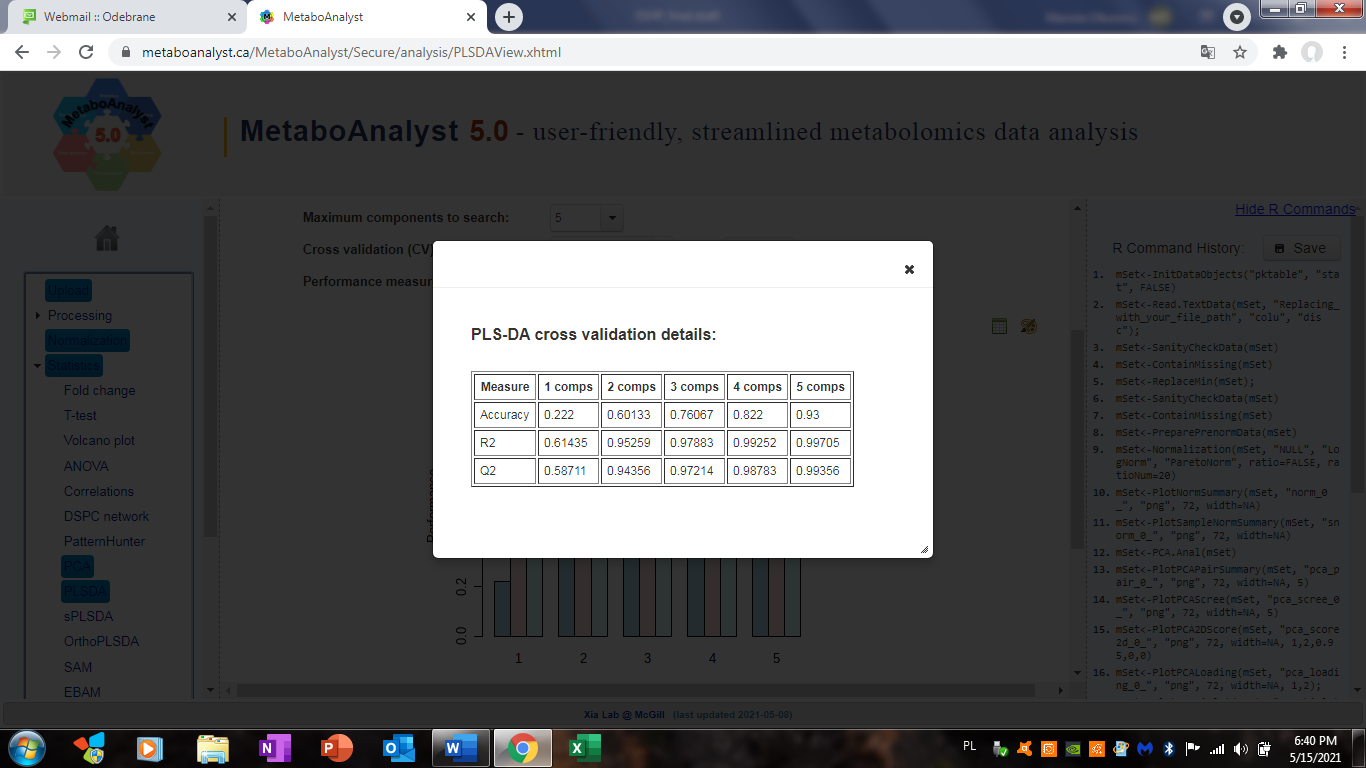


**HEART**

**ESI-**

**ESI+**

**D**

**C**


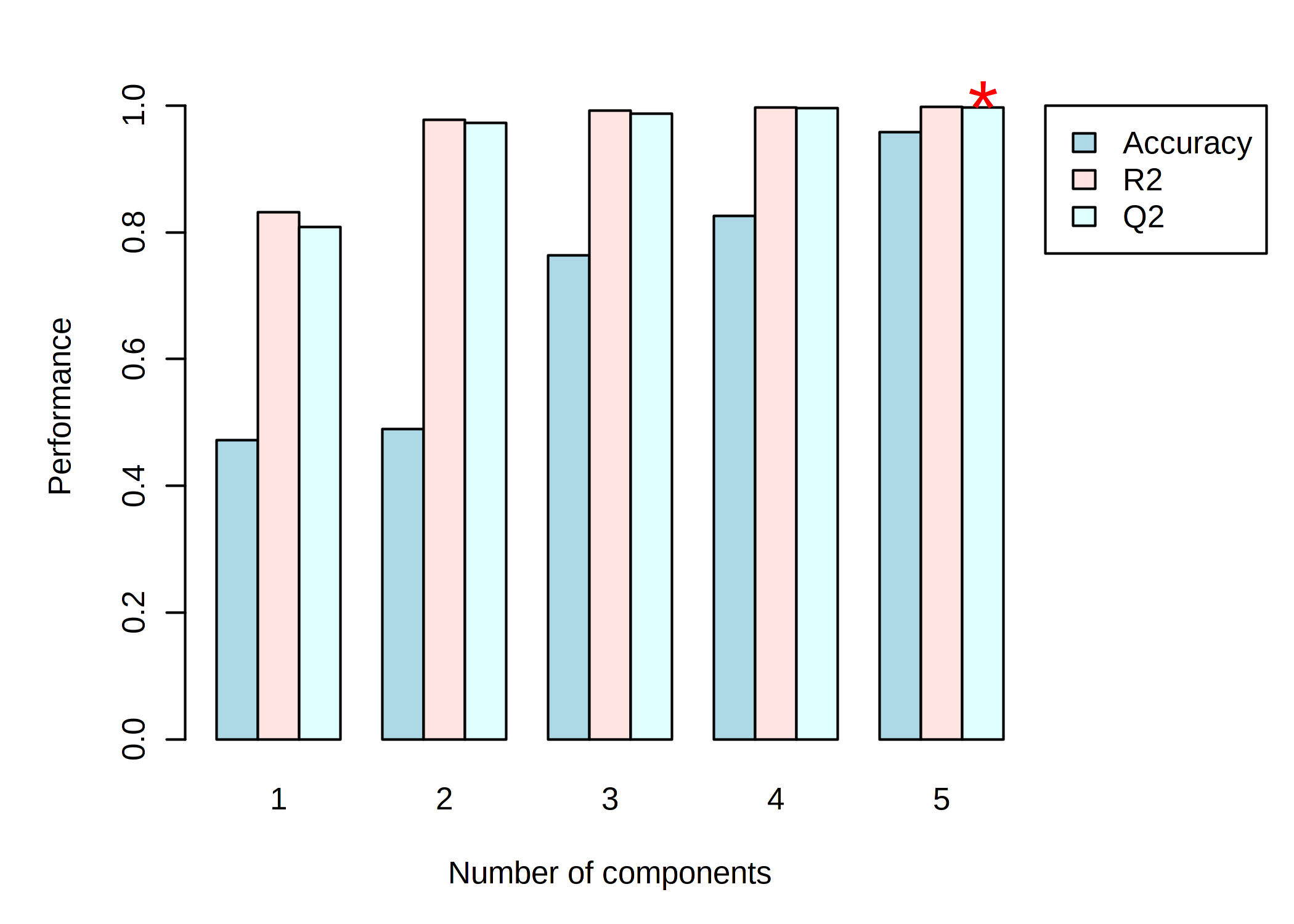

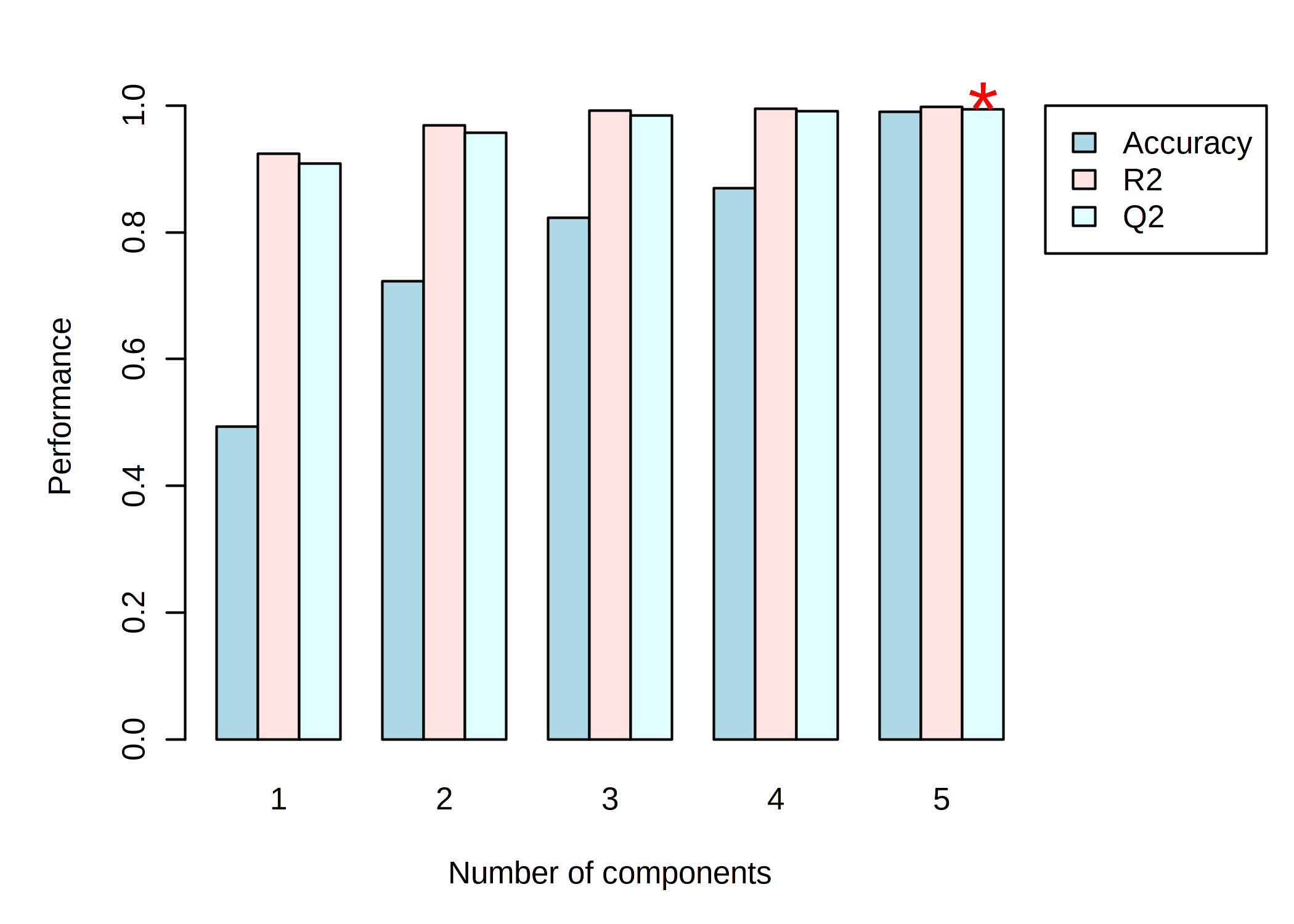


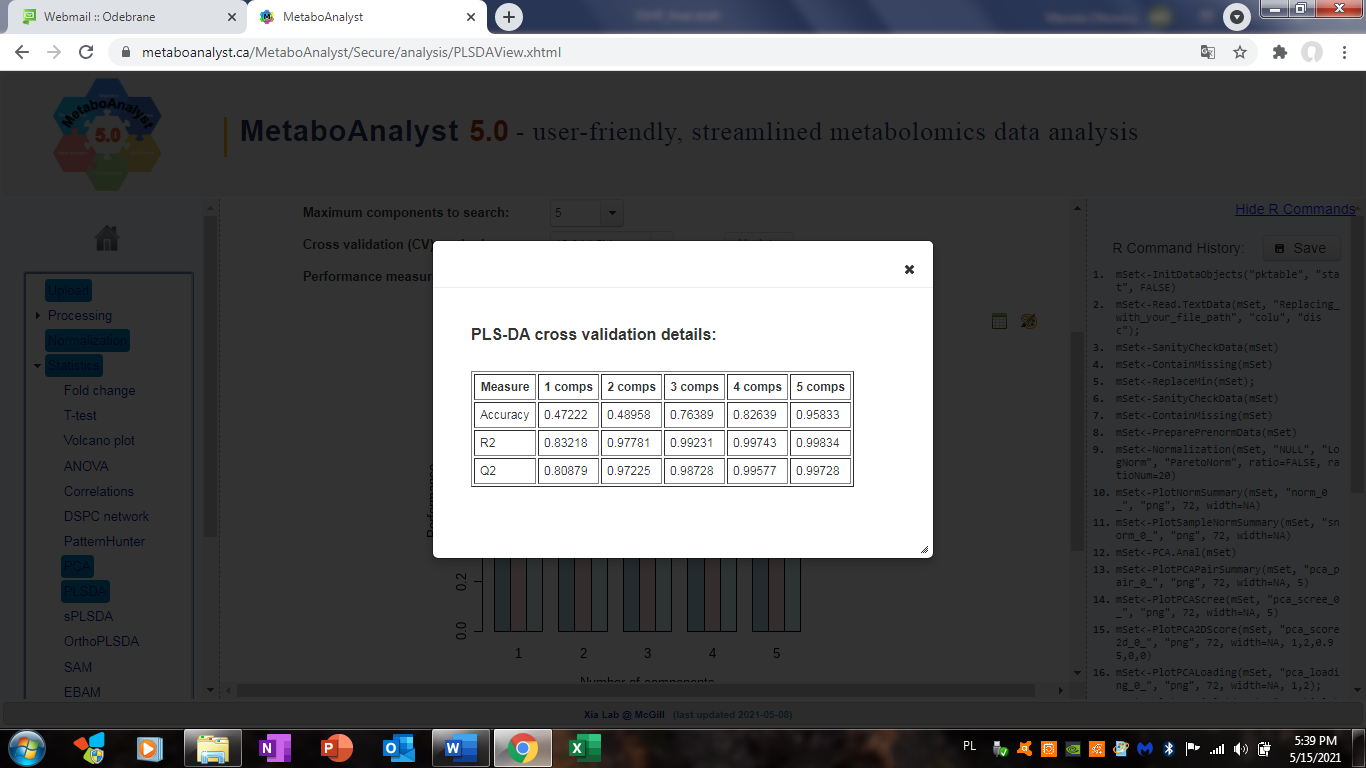

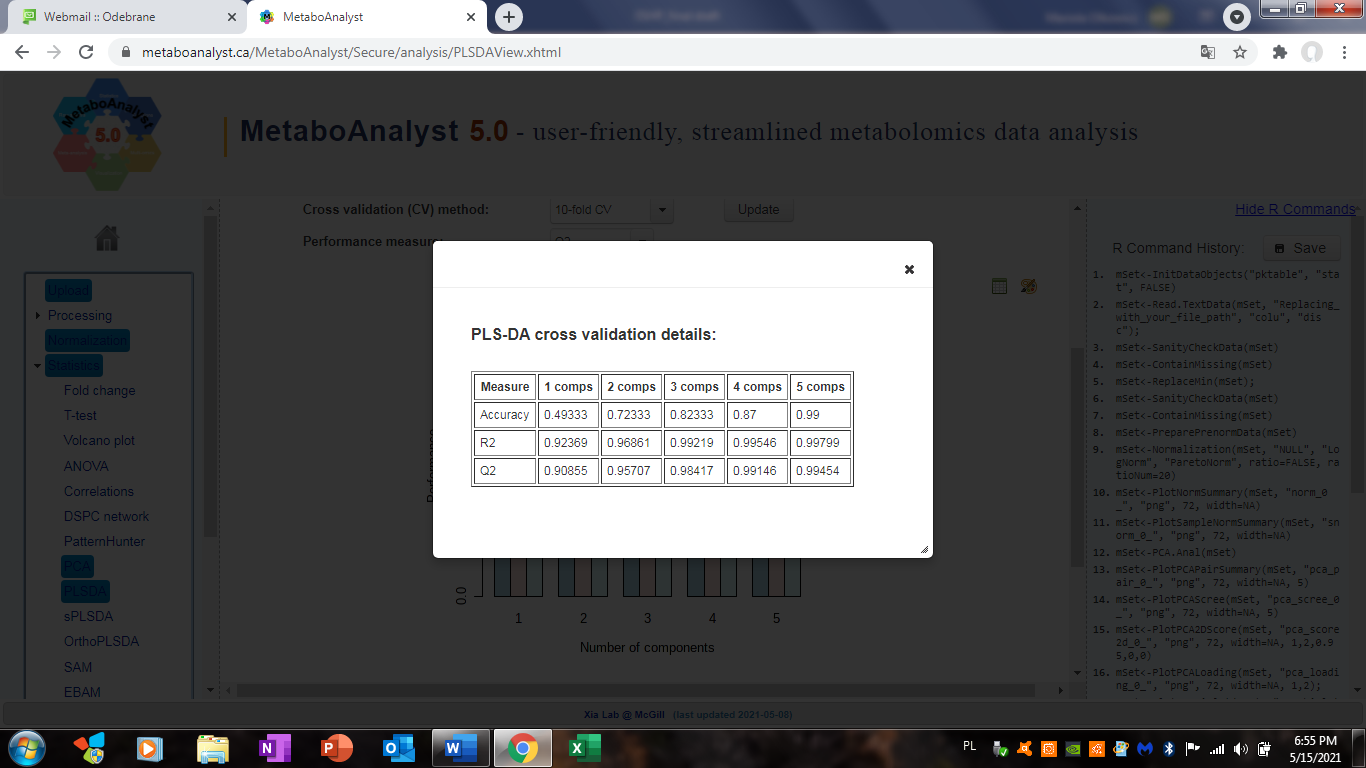


**Supplementary Fig. 15**. Cross-validated predictive residuals (LOOCV) that were used for testing the reliability of the PLS-DA models (**A**, **B** – for 5 explored groups (*in vivo*, ESHP), **C**, **D** – for 4 explored groups (ESHP)). The quality of the models was assessed by the cumulative modeled variation in the X and Y matrix (R2 X and R2 Y) and the cross-validated predictive ability Q2 (cum) values. The 5-component cross-validation measures for R2Xcum of > 0.99 and the Q2cum values of > 0.99 were excellent proving good fit to the data and high predictive ability.

**ESI+**

**ESI+**

**B**

**A**


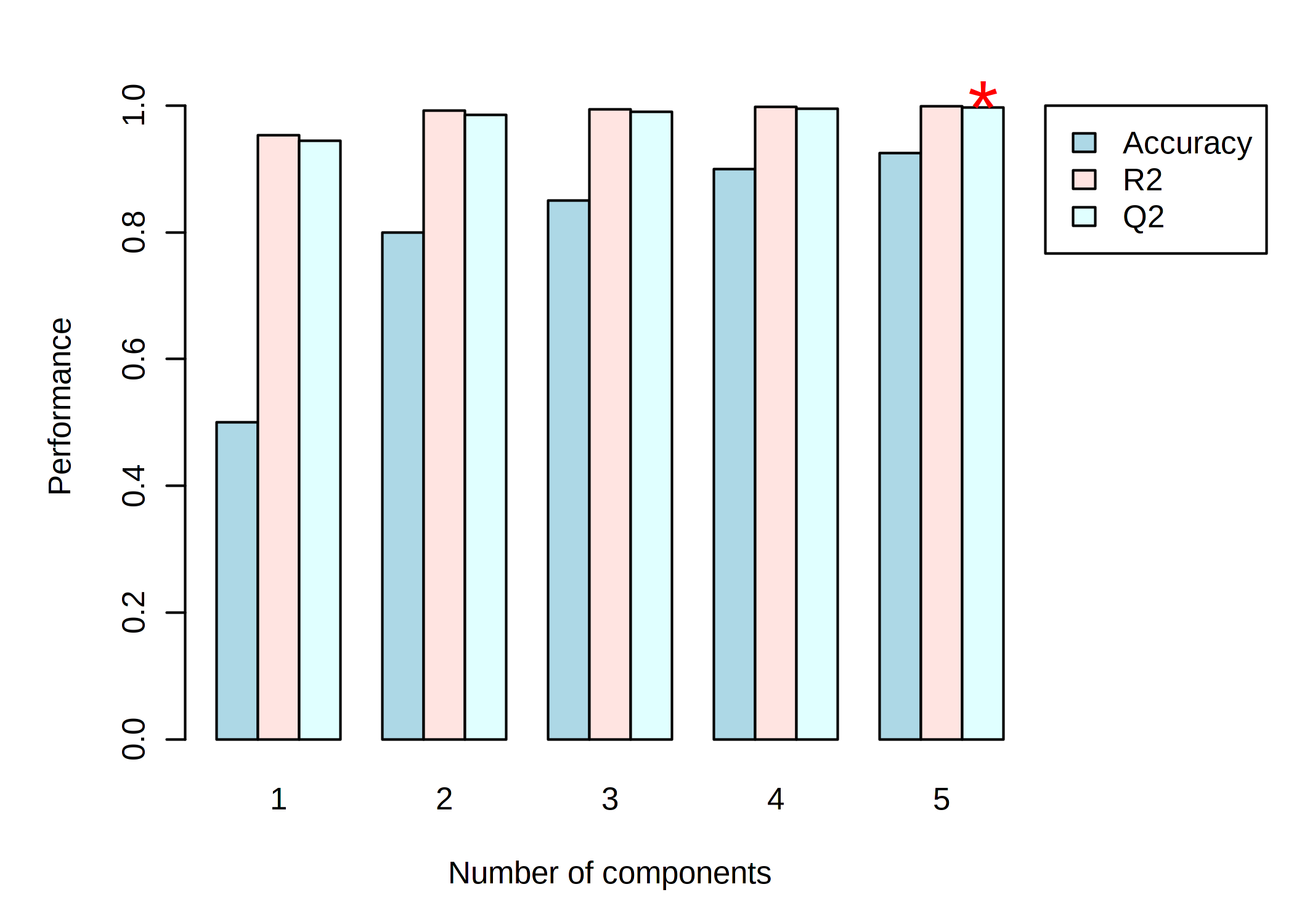

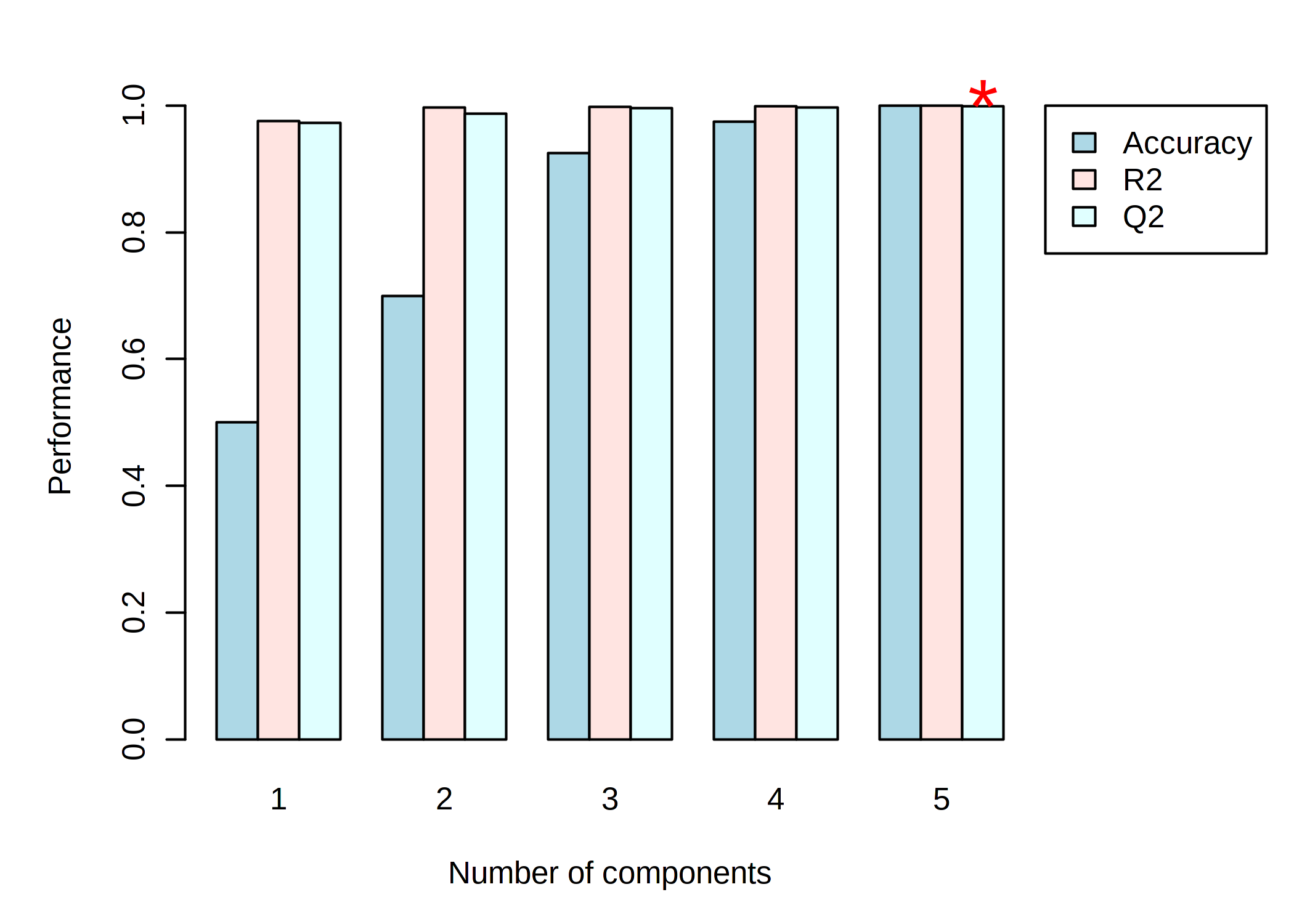


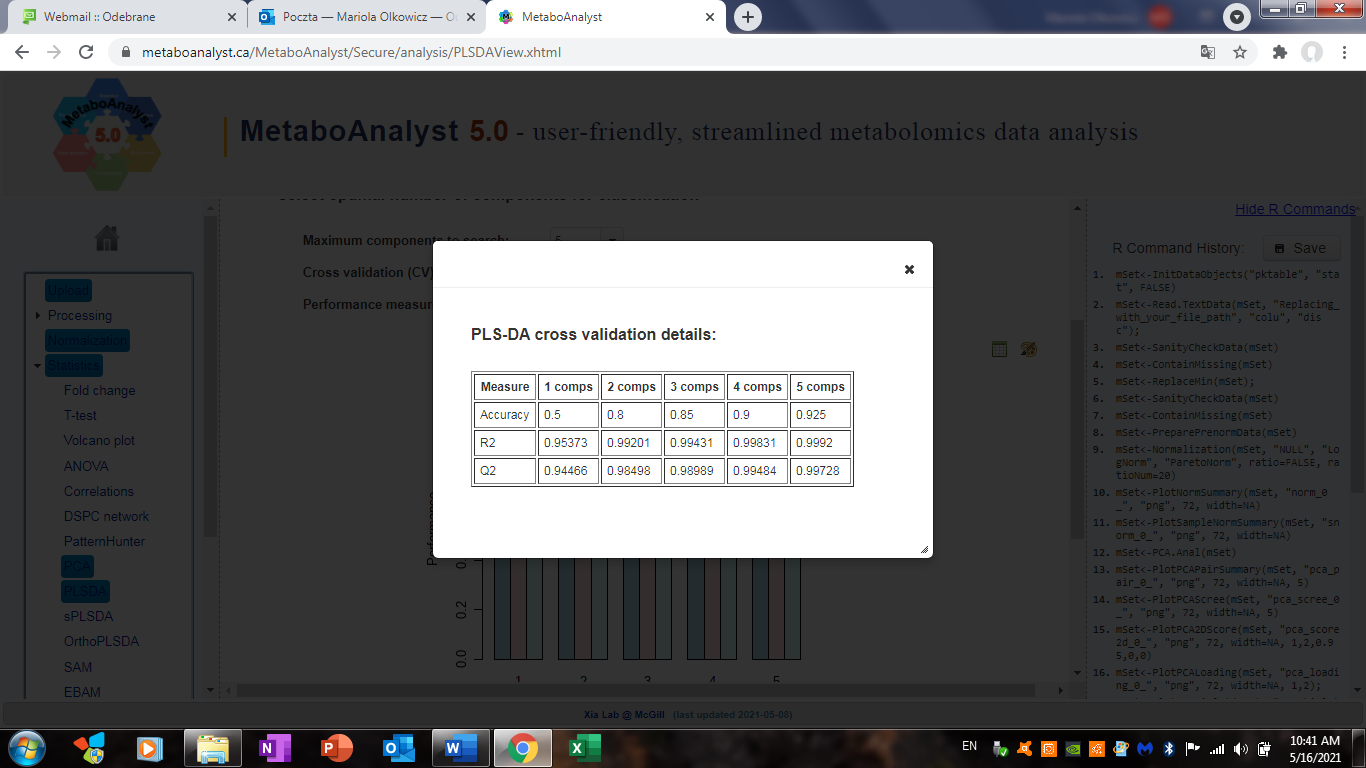

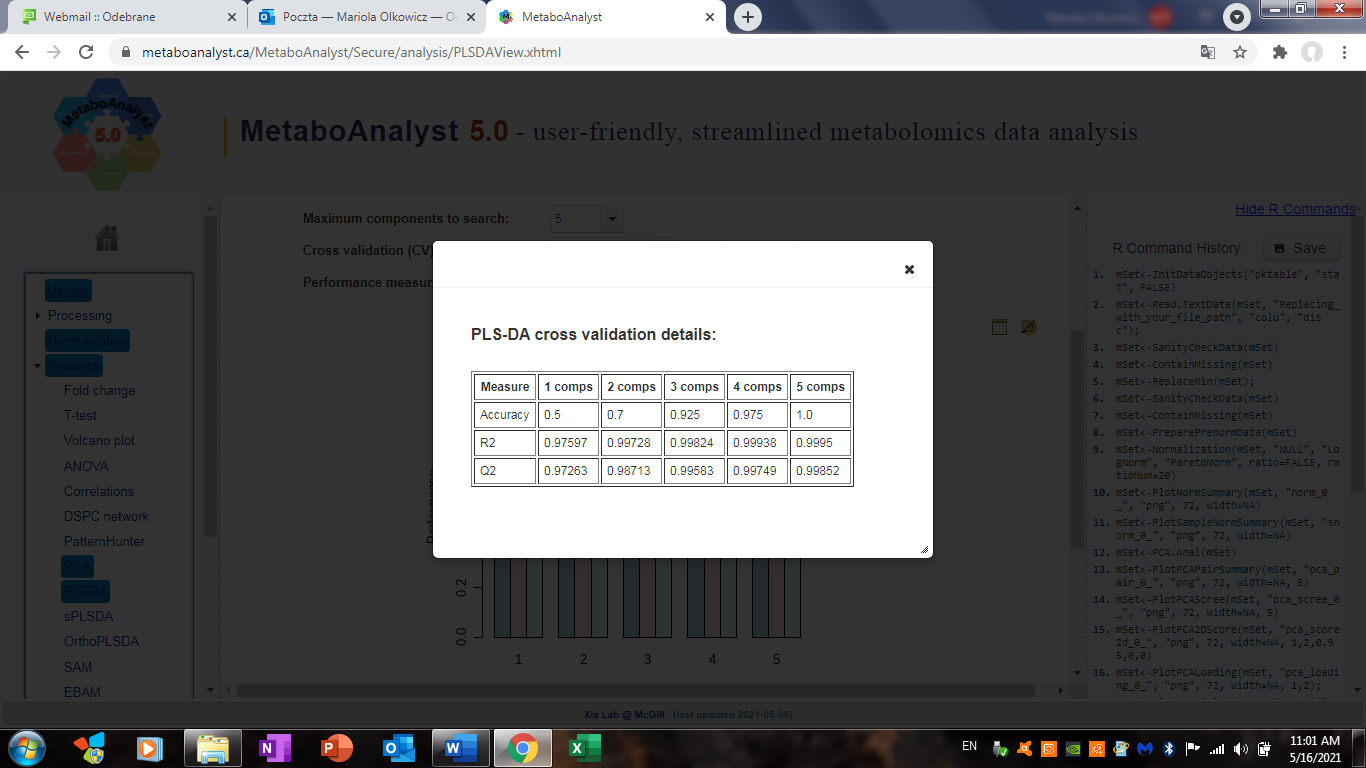


**PERFUSATE**

**ESI-**

**ESI-**

**D**

**C**


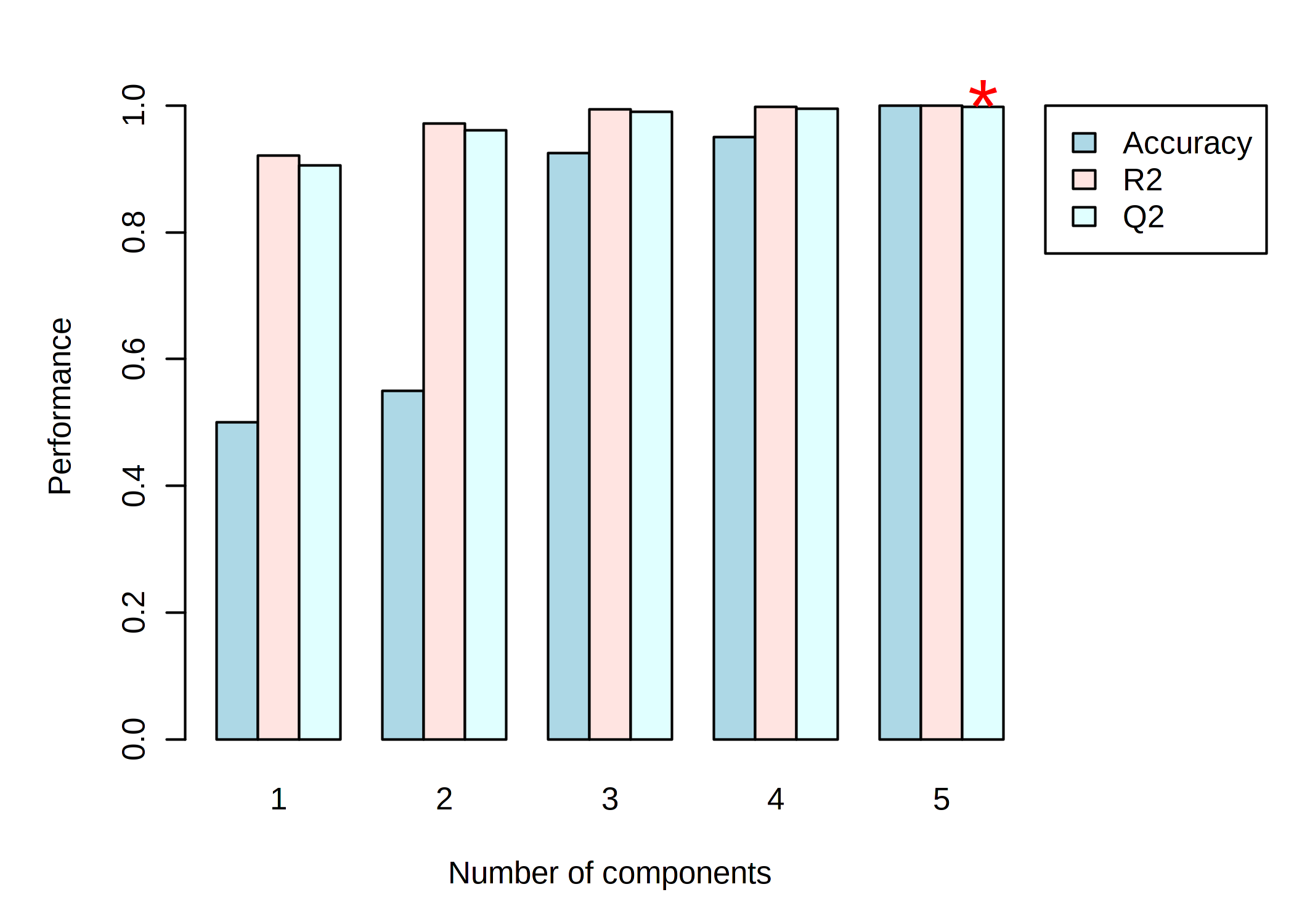

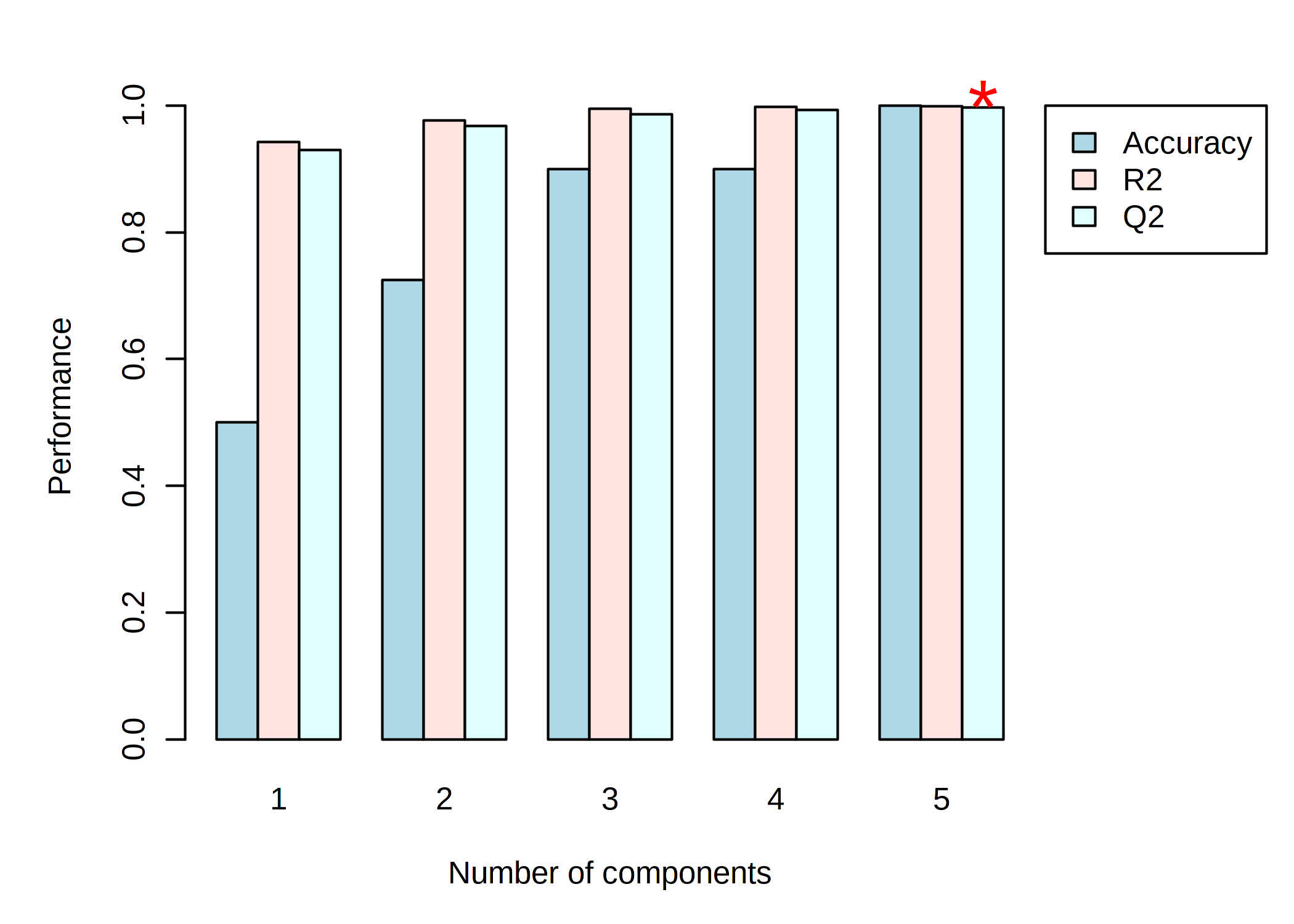


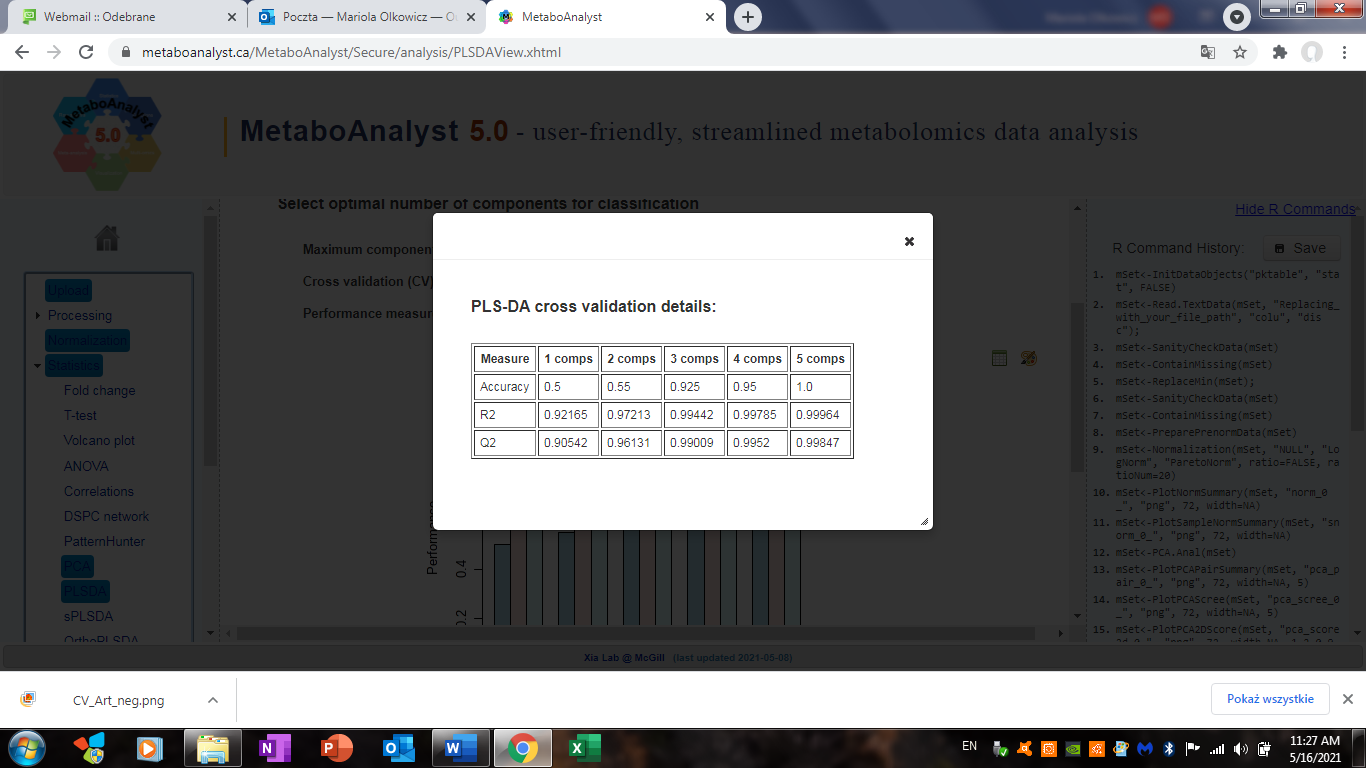

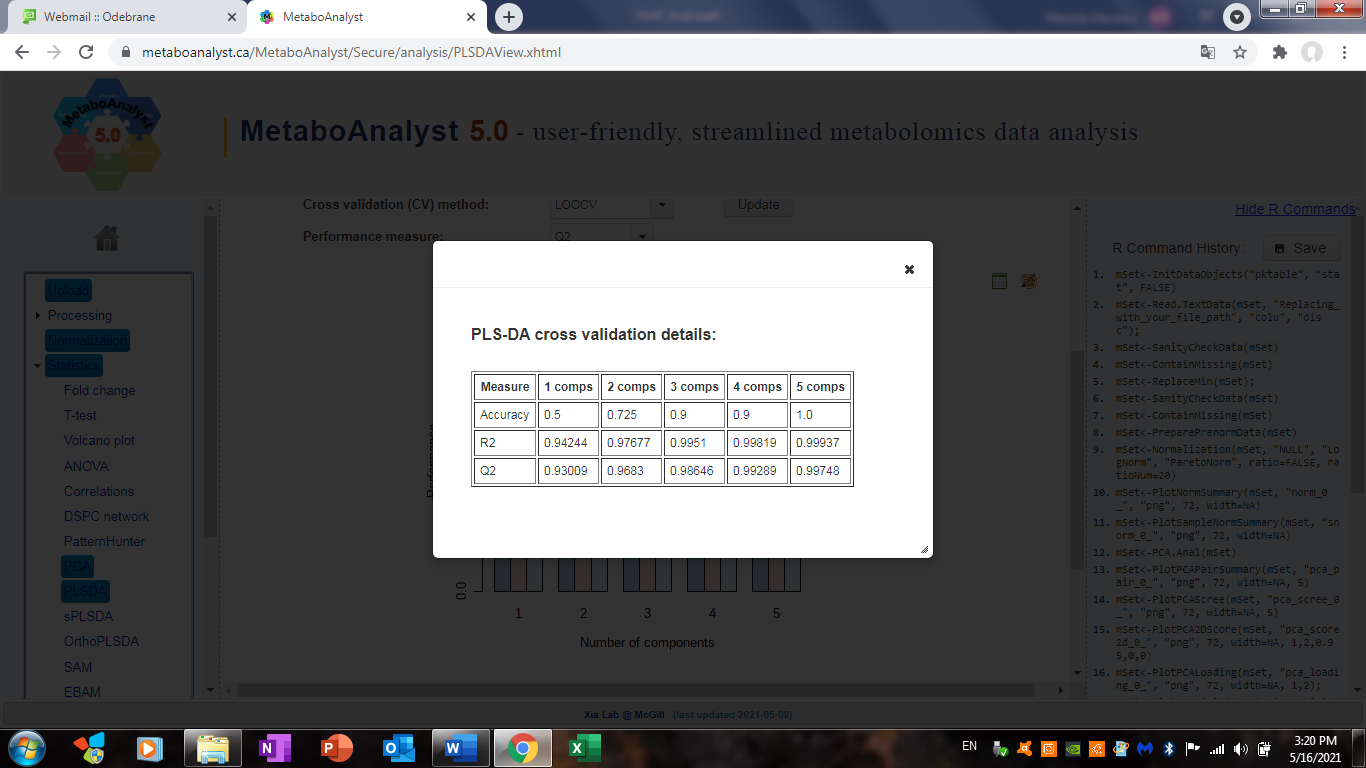


**Supplementary Fig. 16.** Bar plots showing the three performance measures for the PLS-DA models created (prediction accuracy, R^2^ and Q^2^) using different number of components. The asterisk indicates the best values of the indicated measures proving validity of the models/data. **A**, **C** – arterial (perfusate) samples, **B**, **D** – venous (perfusate) samples.

Perfusate sampling – C18 fibers

1^st^ clinical case

**A** Arterial samples **B** Venous samples

**ESI+**


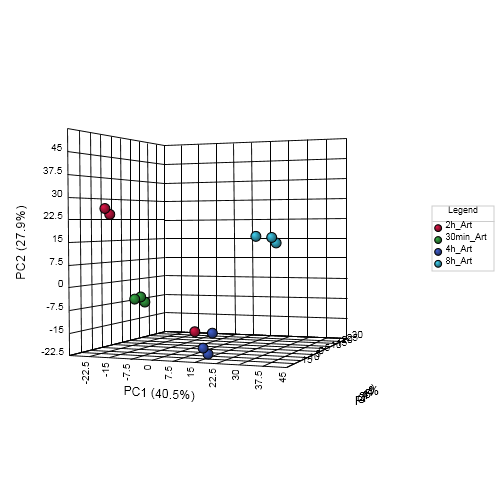

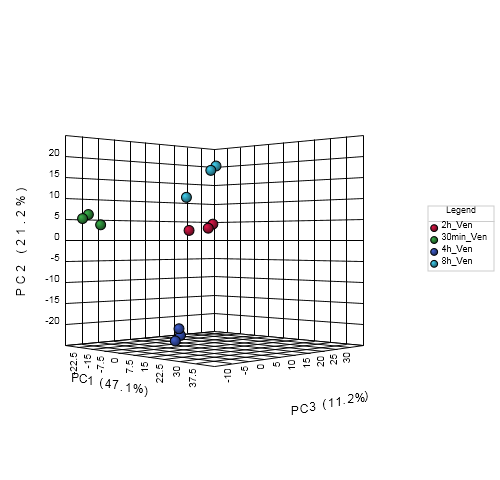


**C** **D**

**ESI-**


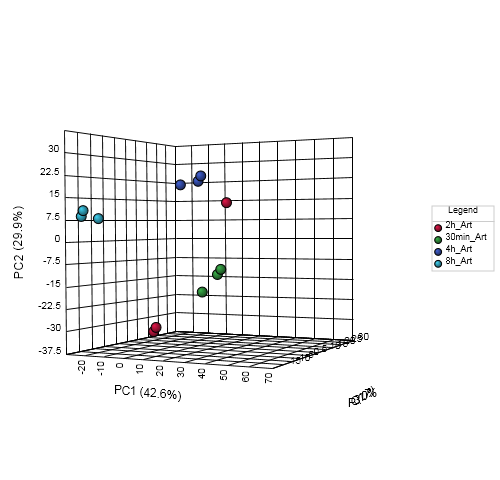

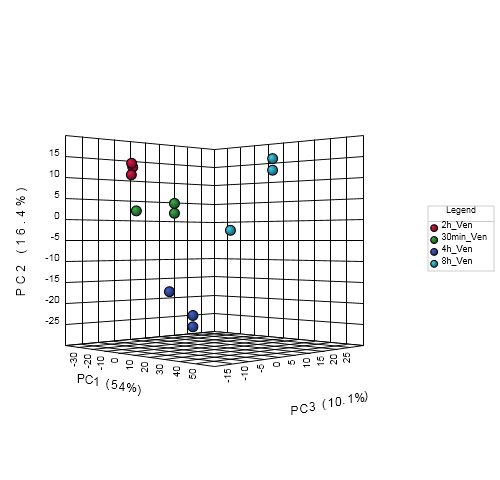


**Supplementary Fig. 17.** PCA score plots for metabolites detected in perfusate extracts collected during the 1^st^ clinical ESHP with the use of C18 SPME probes. The plots were generated for features/metabolites detected in ESI+ (**A**, **B**) and ESI- (**C**, **D**)/RP mode. **Green**—arterial/venous perfusate samples taken at 30 min of ESHP; **red**—arterial/venous perfusate samples taken at 2 h of ESHP; **dark blue**—arterial/venous perfusate samples taken at 4 h of ESHP; **light blue**—arterial/venous perfusate samples taken at 8 h of ESHP.

Perfusate sampling – C18 fibers

2^nd^ clinical case

**A** Arterial samples **B** Venous samples

**ESI+**


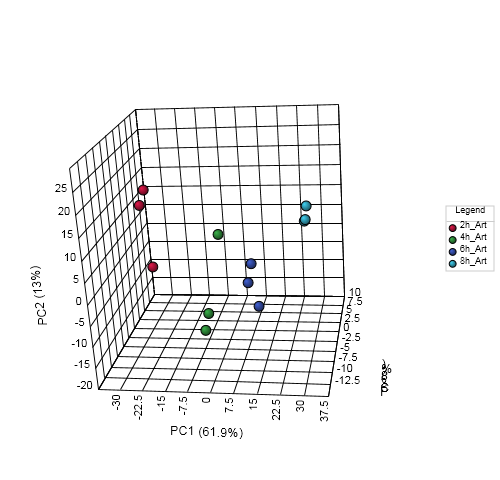

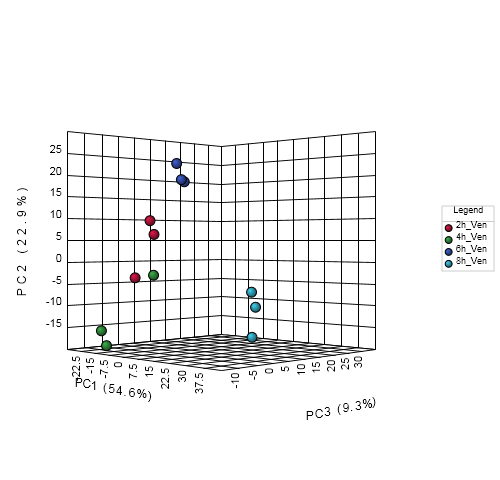


**C** **D**

**ESI-**


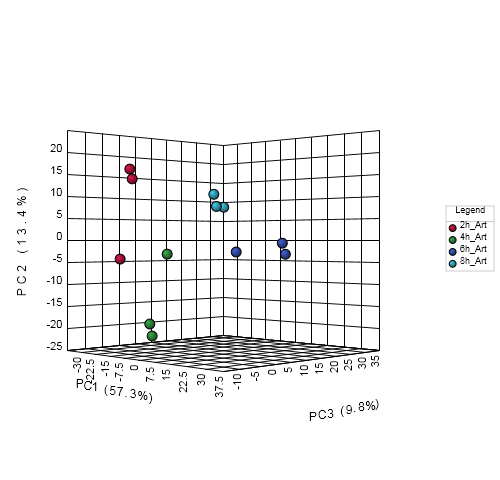

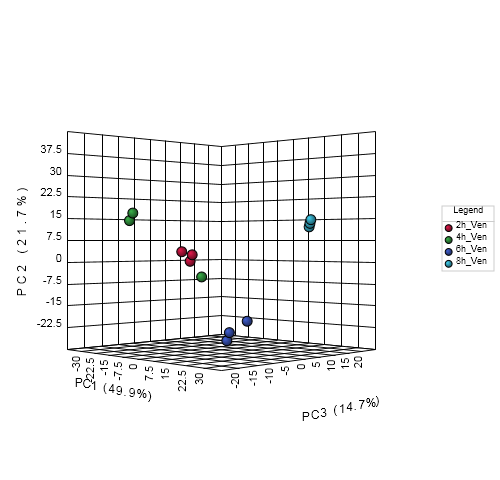


**Supplementary Fig. 18.** PCA score plots for metabolites detected in perfusate extracts collected during the 2^nd^ clinical ESHP with the use of C18 SPME probes. The plots were generated for features/metabolites detected in ESI+ (**A**, **B**) and ESI- (**C**, **D**)/RP mode. **Red**—arterial/venous perfusate samples taken at 2 h of ESHP; **green**—arterial/venous perfusate samples taken at 4 h of ESHP; **dark blue**—arterial/venous perfusate samples taken at 6 h of ESHP; **light blue**—arterial/venous perfusate samples taken at 8 h of ESHP.

**Supplementary Table 1.** Differentially enriched and significant metabolites at specific time points during porcine ESHP between 15 min and 8 h of perfusion. The indicated changes refer to metabolic alterations in perfused hearts. **Pos**: positive mode. **Neg**: negative mode.

| **METABOLITES*** | **m/z** | **RT (min)** | **Adduct** | **Ion mode** | **†Level** | **VIP** | **Ratio‡** | | | | **Metabolic pathway** | |
| --- | --- | --- | --- | --- | --- | --- | --- | --- | --- | --- | --- | --- |
|  |  |  |  |  |  |  | **1.5h/15min** | **4h/1.5h** | **8h/4h** | **8h/15min** |  |  |
| **HEART**  *Chondroitin sulfate E*  *8-Isoprostaglandin E2/8-iso-15-*  *keto-PGF2a*  *15-Deoxy-d-12,14-PGJ_2_*  *Resolvin D1/D2*  *Trihydroxyoctadecenoic acid*  *(two isomers)***  *Ubiquinone-1*  *Hypoxanthine*  *Inosine*  *Adenosine*  *5-Methyldeoxycytidine*  *Butyrylcarnitine*  *Octanoylcarnitine*  *Retinoyl β-glucuronide*  *All-trans-4-Oxoretinoic acid*  *Monoacylglycerol (24:6)*  *Phosphatidylserine (36:5)*  *Diacylglycerol (34:1)*  *Diacylglycerol (38:7)*  *Diacylglycerol (40:8)*  *L-Histidine*  *Cyclic 3-Hydroxymelatonin*  *S-Cysteinosuccinic acid*  *Beta-Citryl-L-glutamic acid*  *Corticosterone*  *11-Dehydrocorticosterone*  *Glycerophosphoethanolamine*  *Dodecanedioic acid*  *Stearoylcarnitine*  *6-Keto-decanoylcarnitine*  *3-Hydroxytetradecanedioic acid*  *Trihydroxyoctadecenoic acid*  *(two isoforms)**  *Ubiquinone-1*  *1-Methylhistamine*  *8-Oxo-dGMP*  *Undecanedioic acid*  *Diacylglycerol (38:7)*  *1-Octen-3-yl glucoside*  *Lysophosphatidic acid (16:0)*  *8-iso-13,14-dihydro-15-keto-PGF2a (8-isoprostane metabolite)*  *20-Hydroxy-PGF2a*  *Pantothenic acid*  *18-Hydroxycorticosterone* | 330.1734  416.2428  317.2111  377.2323  331.2479  331.2479  251.1279  137.0458  269.0881  268.1041  320.0414  232.1545  288.2170  477.2483  297.185  431.3156  820.4538  339.2935  358.2095  378.2251  156.0768  331.1768  365.0664  349.0931  311.2005  327.1953  243.0791  229.1443  464.3148  346.2001  273.1709  329.2336  329.2336  249.1132  271.1665  241.0312  197.1178  361.2236  289.1658  409.2362  353.2337  369.2284  475.1716  421.2235 | 22.05  15.85  16.39  14.88  16.99  17.86  19.59  1.59  5.34  8.96  2.2  16.13  21.68  16.11  15.85  20.23  15.2  19.46  15.85  15.85  1.83  22.06  1.83  1.83  15.86  15.86  17.61  19.55  21.56  15.9  14.26  17.43  17.87  19.38  14.2  14.52  17.51  13.43  14.6  23.75  16.2  14.9  16.45  14.99 | [2M+2H+3H_2_O]+  [M+ACN+Na]+  [M+H]+  [M+H]+  [M+H]+  [M+H]+  [M+H]+  [M+H]+  [M+H]+  [M+H]+  [M+2K+H]3+  [M+H]+  [M+H]+  [M+H]+  [M+H-H2O]+  [M+H]+  [M+K]+  [M+2ACN+2H]2+  [M+2K]2+  [M+2K]2+  [M+H]+  [M+2ACN+H]+  [3M+H+NH4]2+  [2M+3H_2_O+2H]2+  [M+H-2H2O]+  [M+H-H2O]+  [2M+3H_2_O+2H]2+  [M-H]-  [M-2H+K]-  [2M-H+Cl]2-  [M-H]-  [M-H]-  [M-H]-  [M-H]-  [2M-2H+Na]-  [2M-3H]3-  [M-H2O-H]-  [M+2Cl]2-  [M-H]-  [M-H]-  [M-H]-  [M-H]-  [2M-2H+K]-  [M+CH3COO]- | Pos  Pos  Pos  Pos  Pos  Pos  Pos  Pos  Pos  Pos  Pos  Pos  Pos  Pos  Pos  Pos  Pos  Pos  Pos  Pos  Pos  Pos  Pos  Pos  Pos  Pos  Pos  Neg  Neg  Neg  Neg  Neg  Neg  Neg  Neg  Neg  Neg  Neg  Neg  Neg  Neg  Neg  Neg  Neg | 5  4  4  4  4  4  2  1  1  1  5  2  2  5  5  3  3  3  3  3  1  5  5  5  1  4  5  5  2  5  5  4  4  2  4  4  5  3  5  3  5  5  5  4 | 1.9  2.1  2.1  2.6  2.3  2.3  2.4  1.5  2.5  1.5  1.8  1.5  2.3  2.9  1.7  2.4  1.9  1.6  1.5  1.6  1.5  1.9  1.5  1.5  1.7  1.7  1.5  3.1  1.8  1.6  2.1  2.9  2.8  2.8  2.6  2.2  1.8  1.8  1.8  1.6  1.7  1.6  1.6  1.7 | 11.42  4.48  10.04  3.66  0.69  0.54  2.61  1.26  0.32  0.46  0.81  0.16  0.12  1.54  1.98  2.13  2.94  2.85  1.91  1.91  0.76  8.26  0.67  0.71  2.01  1.91  0.81  3.49  0.73  1.84  6.35  0.58  0.52  3.29  0.59  2.25  1.64  1.83  17.30  0.52  3.48  4.71  1.59  2.41 | 2.24  1.80  2.03  2.70  0.27  0.23  2.92  1.30  0.25  0.79  0.64  0.61  0.43  3.69  1.45  2.44  1.59  1.47  1.35  1.39  0.83  2.38  0.83  0.86  1.27  1.48  0.76  5.57  1.40  1.44  2.29  0.17  0.20  3.75  0.55  2.06  1.84  1.86  1.94  0.62  0.83  0.83  1.31  1.84 | 1.36  3.41  2.35  3.09  0.77  0.86  2.15  1.07  1.17  0.81  0.58  1.05  0.27  2.60  1.85  2.42  1.84  1.71  1.82  1.78  0.64  1.38  0.50  0.48  1.96  1.82  0.54  1.68  2.69  1.66  1.35  0.73  0.71  1.98  0.23  1.86  1.39  1.46  1.08  0.80  3.21  2.39  2.05  2.94 | 34.90  27.53  47.99  30.60  0.14  0.11  16.42  1.75  0.09  0.29  0.30  0.10  0.02  14.75  5.29  12.57  8.64  7.16  4.68  4.72  0.40  27.04  0.28  0.29  5.03  5.16  0.34  32.71  2.75  4.42  19.63  0.07  0.07  24.39  0.07  8.60  4.20  4.97  36.39  0.26  9.27  9.33  4.26  13.02 | *Inflammatory response*  *Arachidonic acid metabolism*    *Arachidonic acid metabolism*  *Docosahexaenoic acid metabolism*  *Regulation of prostaglandin*  *synthesis*  *Electron transport chain (ETC)*  *Purine metabolism*  *Purine metabolism*  *Purine metabolism*  *Regulation of gene expression*  *FA β-oxidation*  *FA β-oxidation*  *Retinol metabolism*  *Retinol metabolism*  *Lipid metabolism*  *Lipid metabolism*  *Lipid metabolism*  *Lipid metabolism*  *Lipid metabolism*  *Amino acid metabolism*  *ROS generation*  *Krebs cycle*  *Krebs cycle*  *Aldosterone synthesis*  *Corticosterone metabolism*  *Phospholipid biosynthesis*  *FA ω-oxidation*  *FA β-oxidation*  *FA β-oxidation*  *FA ω-oxidation*  *Regulation of prostaglandin*  *synthesis*  *Electron transport chain*  *Histidine metabolism*  *ROS generation*  *Lipid metabolism*  *Lipid metabolism*  *Lipid metabolism*  *Intercellular lipid signaling*  *Arachidonic acid metabolism/*  *Oxidative stress*  *Immune resp./vasocontraction*  *CoA biosynthesis*  *Aldosterone synthesis* |  |

* metabolites were organized according to the type of ionization mode and pathway involved;

** 9,10,13-Trihydroxyoctadec-11-enoic acid and 9,12,13-Trihydroxyoctadec-10-enoic acid;

**†** Levels of identification: **5** – annotation using xMSannotator Integrative Scoring Algorithm (details on this tool can be found in the ‘Data Handling and Statistical Analysis’ section; only features with medium-to-high confidence annotations were included); **4** – found in 2 ionization modes; **3** – putatively characterized metabolite or lipid class; **2** – MS/MS spectrum match with database (putatively annotated metabolite), **1** – chromatographic retention time (Rt), m/z, mass fragmentation pattern match with authentic standard (high-confidence metabolite identification);

**‡** Ratios of the average MS ion intensities (peak areas) between particular groups studied. All the discriminant metabolites or lipid species involved in this table met significance threshold of VIP≥1.5 and/or FDR-adjusted P<0.05.

**Supplementary Table 2.** Differentially enriched and significant metabolites at specific time points during porcine ESHP between 15 min and 8 h of perfusion. The indicated changes refer to metabolic alterations in perfusate samples. **Pos**: positive mode. **Neg**: negative mode.

| **METABOLITES*** | **m/z** | **RT (min)** | **Adduct** | **Ion mode** | **Level** | **VIP** | **Ratio‡** | | | | **Metabolic pathway** | |
| --- | --- | --- | --- | --- | --- | --- | --- | --- | --- | --- | --- | --- |
|  |  |  |  |  |  |  | **1.5h/15min** | **4h/1.5h** | **8h/4h** | **8h/15min** |  |  |
| **PERFUSATES**  *Resolvin D1/D2*  *8-Isoprostaglandin E2/8-iso-15-keto-PGF2a*  *15-Deoxy-delta-12,14-PGJ2*  *Trihydroxyoctadecenoic acid*  *(two isomers)***  *Inosine*  *Adenosine*  *(A)symmetric dimethylarginine*  *Isoxanthopterin*  *Retinoyl β-glucuronide*  *18-Hydroxyretinoic acid*  *Ubiquinone-1*  *Monoacylglycerol (24:6)*  *Diacylglycerol (38:1)*  *Diacylglycerol (40:8)*  *LysoPE(P-16:0)*  *LysoPC(P-16:0)*  *L-Cystine*  *Corticosterone*  *11-Dehydrocorticosterone*  *Ubiquinone-1*  *Thromboxane B2*  *Trihydroxyoctadecenoic acid*  *(two isomers)***  *Stearoylcarnitine*  *Octadecenylcarnitine*  *Dodecanedioic acid*  *3-Hydroxytetradecanedioic acid*  *Docosapentaenoic acid*  *Docosahexaenoic acid*  *Monoacylglycerol (24:6)*  *LysoPE (P-16:0)*  *Lysophosphatidic acid (16:0)*  *8-Oxo-dGMP*  *Methionine sulfoxide*  *11-Dehydrocorticosterone*  *18-Hydroxycorticosterone* | 341.2111  416.2428  281.1901  331.248  331.248  269.0881  268.1041  323.2008  180.0512  477.2483  281.1902  251.1279  431.3156  367.3248  378.2251  438.2979  480.3449  241.0311  311.2005  327.1952  249.1132  369.2284  329.2336  329.2336  464.3148  462.2994  229.1443  273.1709  329.2488  327.2332  429.3011  436.2835  409.2362  241.0312  329.0853  343.1916  421.2235 | 14.85  15.87  14.84  17.0  17.87  5.43  8.96  14.84  13.4  16.13  15.87  19.6  20.23  20.99  15.87  20.5  21.03  1.22  15.89  15.88  19.37  14.89  17.0  17.87  21.56  20.79  19.54  14.24  24.03  23.75  20.26  20.47  23.75  14.51  10.84  15.86  14.99 | [M+H-2H2O]+  [M+ACN+Na]+  [M+H-2H2O]+  [M+H]+  [M+H]+  [M+H]+  [M+H]+  [3M+H+K]2+  [M+H]+  [M+H]+  [M+H-2H2O]+  [M+H]+  [M+H]+  [M+2ACN+2H]2+  [M+2K]2+  [M+H]+  [M+H]+  [M+H]+  [M+H-2H2O]+  [M+H-H2O]+  [M-H]-  [M-H]-  [M-H]-  [M-H]-  [M+K-2H]-  [M+K-2H]-  [M-H]-  [M-H]-  [M-H]-  [M-H]-  [M-H]-  [M-H]-  [M-H]-  [2M-3H]-  [2M-H]-  [M-H]-  [M+CH3COO]- | Pos  Pos  Pos  Pos  Pos  Pos  Pos  Pos  Pos  Pos  Pos  Pos  Pos  Pos  Pos  Pos  Pos  Pos  Pos  Pos  Neg  Neg  Neg  Neg  Neg  Neg  Neg  Neg  Neg  Neg  Neg  Neg  Neg  Neg  Neg  Neg  Neg | 4  4  4  4  4  1  1  1  5  5  5  2  3  3  3  3  3  1  1  4  2  5  4  4  2  2  5  5  5  5  3  3  3  4  2  4  4 | A/V***  3.8/3.7  2.4/2.4  2.2/2.2  2.1/1.5  2.1/1.7  3.3/2.9  1.5/1.5  2.7/2.5  1.9/2.1  2.1/2.2  1.5/1.6  1.8/2.1  1.8/1.9  1.5/1.6  1.5/1.6  1.6/1.8  1.5/1.6  1.5/1.7  1.5/1.6  1.5/1.6  2.2/2.5  1.9/2.4  2.1/1.8  2.8/2.5  2.0/2.3  1.9/2.1  2.6/2.6  2.1/2.0  1.9/1.5  2.1/1.8  2.0/2.1  1.7/1.9  2.1/1.8  2.0/1.9  1.6/1.9  1.5/1.6  1.9/1.8 | A/V  131.2/124.4  4.91/ 6.60  23.06/30.13  1.57/0.92  1.17/1.07  0.29/0.30  0.15/0.15  2.23/3.02  30.25/ 9.75  1.42/ 35.73  1.77/ 1.62  18.61/13.19  4.64/4.17  1.46/1.17  1.94/1.68  1.68/1.08  1.08/ 0.83  0.88/0.91  2.48/1.89  2.55/1.97  9.49/5.66  5.17/3.02  1.30/1.09  1.25/0.90  2.77/1.04  2.30/1.91  3.46/4.05  6.43/9.05  0.92/0.48  0.72/0.45  5.38/2.83  1.76/1.10  0.70/0.46  5.95/4.42  0.94/0.76  1.91/1.67  3.30/2.48 | A/V  3.94/4.40  4.20/4.42  4.72/5.63  0.21/0.33  0.20/0.19  0.02/0.04  0.30/1.36  8.92/8.80  1.26/1.98  4.29/5.22  1.79/1.91  2.36/3.60  2.18/2.88  1.87/2.49  1.83/2.06  1.68/2.34  1.33/1.84  1.79/2.91  1.46/1.86  1.47/2.11  3.37/4.72  2.72/5.10  0.23/ 0.28  0.13/0.19  1.72/2.44  2.29/3.16  7.56/8.29  3.52/3.73  0.19/0.27  0.15/ 0.17  2.42/3.21  1.81/1.98  0.14/0.12  2.90/3.08  2.11/3.0  1.42/1.62  1.65/1.97 | A/V  2.79/3.87  3.68/5.40  2.65/2.83  0.54/0.75  0.56/0.72  0.34/0.34  3.24/0.31  3.47/3.47  1.86/1.66  2.69/2.97  1.66/2.16  2.32/3.22  2.56/2.67  1.66/1.54  1.73/1.98  2.32/2.80  2.11/2.48  1.55/1.72  1.74/2.18  2.09/2.08  3.11/3.64  2.38/2.35  0.67/0.64  0.30/0.30  4.43/5.33  2.22/2.20  2.21/2.55  1.81/1.90  1.14/1.23  1.16/1.49  2.75/2.74  2.52/3.34  1.37/2.0  1.98/1.90  2.19/2.18  1.58/1.99  2.81/2.99 | A/V  1443/2120  75.83/157.5  288.5/481.1  0.17/0.23  0.13/0.14  0.002/0.004  0.15/0.06  69.07/92.29  70.89/31.96  16.41/553.9  5.26/ 6.66  102.1/153.0  25.81/32.04  4.56/4.51  6.16/6.83  6.55/7.10  3.04/3.80  2.43/4.56  6.30/7.64  7.83/8.64  99.45/97.05  33.39/36.11  0.20/0.19  0.05/0.05  21.17/13.58  11.67/13.25  57.75/85.77  40.95/64.02  0.20/0.16  0.13/0.11  35.83/24.86  8.01/7.24  0.14/0.11  34.27/25.80  4.36/4.98  4.29/5.37  15.29/14.61 | *Docosahexaenoic acid metabolism*  *Arachidonic acid metabolism*  *Arachidonic acid metabolism*  *Regulation of prostaglandin*  *synthesis*  *Purine metabolism*  *Purine metabolism*  *NO generation*  *NO generation/BH_4_ degradation*  *Retinol metabolism*  *Retinol metabolism*  *Electron transport chain (ETC)*  *Lipid metabolism*  *Lipid metabolism*  *Lipid metabolism*  *Lipid metabolism/signaling*  *Lipid metabolism*  *Oxidative stress*  *Aldosterone synthesis*  *Corticosterone metabolism*  *Electron transport chain*  *Inflammation/Edema formation*  *Regulation of prostaglandin syn-*  *thesis*  *FA β-oxidation*  *FA β-oxidation*  *FA ω-oxidation*  *FA ω-oxidation*  *α-Linolenic acid (ALA) metabol.*  *Remodeling of the mitochondrial phospholipidome/ALA metab.*  *Lipid metabolism*  *Lipid metabolism/signaling*  *Intercellular lipid signaling*  *ROS generation*  *ROS generation*  *Corticosterone metabolism*  *Aldosterone synthesis* |  |

* metabolites were organized according to the type of ionization mode and pathway involved.

** 9,10,13-Trihydroxyoctadec-11-enoic acid and 9,12,13-Trihydroxyoctadec-10-enoic acid.

*** arterial/venous (perfusate) samples.

Levels of identification: **5** – annotation using xMSannotator Integrative Scoring Algorithm (details on this tool can be found in the ‘Data Handling and Statistical Analysis’ section; only features with medium-to-high confidence annotations were included); **4** – found in 2 ionization modes; **3** – putatively characterized metabolite or lipid class; **2** – MS/MS spectrum match with database (putatively annotated metabolite), **1** – chromatographic retention time (Rt), m/z, mass fragmentation pattern match with authentic standard (high-confidence metabolite identification);

**‡** Ratios of the average MS ion intensities (peak areas) between particular groups studied. All the discriminant metabolites or lipid species involved in this table met significance threshold of VIP≥1.5 and/or FDR-adjusted P<0.05.

**Supplementary Table 3.** Differentially enriched and significant metabolites at specific time points during human ESHP between 30 min and 8 h of perfusion. The indicated changes refer to metabolic alterations in perfused hearts. **Pos**: positive mode. **Neg**: negative mode.

| **METABOLITES*** | **m/z** | **RT (min**) | **Adduct** | **Ion** | **Level** | **Ratio‡** | | | | **Metabolic pathway** |  |
| --- | --- | --- | --- | --- | --- | --- | --- | --- | --- | --- | --- |
|  |  |  |  | mode |  | **†2h/30min**  **††4h/2h** | **4h/2h**  **6h/4h** | **8h/4h**  **8h/6h** | **8h/30min**  **8h/2h** |  | |
| **1^o^ case**  *Glyceraldehyde-3-phosphate*  *Chondroitin sulfate E*  *Resolvin D1/D2*  *8-isoprostaglandin E2/8-iso-15-*  *keto-PGF2a*  *Trihydroxyoctadecenoic acid*  *Ubiquinone-1*  *Hypoxanthine*  *Inosine*  *Adenosine*  *Cyclic 3-Hydroxymelatonin*  *Butyrylcarnitine*  *Phosphatidylserine (32:2)*  *Cardiolipin (82:15)*  *Corticosterone*  *11-Dehydrocorticosterone*  *3-Hydroxytetradecanedioic acid*  *Lysophosphatidic acid (16:0)*  *Phosphatidylglycerol (40:10)*  *Phosphatidylinositol (24:0)*  *Phosphatidylcholine (22:6)*  *L-Cysteinylglycine disulfide*  *18-Hydroxycorticosterone*  **2^o^ case**  *6,15-diketo-13,14-dihydro-prostaglandin F1α*  *PE-Cer(d15:2/20:0(2OH))*  *Chondroitin sulfate E*  *Resolvin D1/D2*  *8-isoprostaglandin E2/8-iso-15-*  *keto-PGF2a*  *Trihydroxyoctadecenoic acid*  *(two isomers)*  *Ubiquinone-1*  *Adenosine*  *Cyclic 3-Hydroxymelatonin*  *Butyrylcarnitine*  *Phosphatidylinositol (28:1)*  *Corticosterone*  *6-Keto-prostaglandin F1α*  *Prostaglandin E1 (PGE1)*  *Hydroxytetradecanedioic acid*  *(two isomers)*  *Phosphatidylglycerol (40:10)*  *L-Cysteinylglycine disulfide*  *Resolvin D1/D2*  *18-Hydroxycorticosterone*  *Phosphatidylserine (34:5)* | 188.0313  330.1734  377.2323  416.2428  331.248  251.1279  137.0458  269.0881  268.1041  331.1768  232.1545  754.4606  521.3432  311.2007  327.196  273.1709  409.236  813.4709  348.193  626.3468  296.0381  421.2235  353.2325  711.505  330.1734  377.2323  416.2429  331.2479  331.2479  251.1279  268.1041  331.1768  232.1545  753.4575  311.2008  369.2284  353.2337  273.1709  273.1709  813.4711  296.0381  435.2388  421.2236  812.4679 | 11.92  22.75  14.82  15.73  17.81  19.57  1.48  5.53  9.15  22.75  16.74  14.82  19.82  15.74  15.74  14.83  23.73  14.85  14.4  20.0  2.38  14.85  14.84  22.21  22.64  14.79  15.71  16.93  17.8  19.55  9.15  22.65  16.68  14.8  15.71  14.83  16.13  14.19  14.83  14.86  2.36  14.86  14.86  14.9 | [M+NH4]+  [2M+2H+3H_2_O]+  [M+H]+  [M+ACN+Na]+  [M+H]+  [M+H]+  [M+H]+  [M+H]+  [M+H]+  [M+2ACN+H]+  [M+H]+  [M+Na]+  [M+2H+Na]3+  [M+H-2H2O]+  [M+H-H2O]+  [M-H]-  [M-H]-  [M-H]-  [M-2H]2-  [M+CH3COO]-  [M-H]-  [M+CH3COO]-  [M+H-H2O]+  [M+Na]+  [2M+2H+3H2O]+  [M+H]+  [M+ACN+Na]+  [M+H]+  [M+H]+  [M+H]+  [M+H]+  [M+2ACN+H]+  [M+H]+  [M+H]+  [M+H-2H2O]+  [M-H]-  [M-H]-  [M-H]-  [M-H]-  [M-H]-  [M-H]-  [M+CH3COO]-  [M+CH3COO]-  [M+CH3COO]- | Pos  Pos  Pos  Pos  Pos  Pos  Pos  Pos  Pos  Pos  Pos  Pos  Pos  Pos  Pos  Neg  Neg  Neg  Neg  Neg  Neg  Neg  Pos  Pos  Pos  Pos  Pos  Pos  Pos  Pos  Pos  Pos  Pos  Pos  Pos  Neg  Neg  Neg  Neg  Neg  Neg  Neg  Neg  Neg | 5  5  4  4  4  2  1  1  1  5  2  3  5  1  4  5  3  3  3  3  5  4  4  3  5  4  4  4  4  2  1  5  2  3  1  5  5  5  5  3  5  4  4  3 | 20.45  4.24  4.75  4.60  0.64  4.55  2.28  1.58  1.19  4.65  2.32  12.47  0.76  1.96  2.14  6.94  0.48  4.05  0.53  0.09  1.14  3.37  9.23  16.88  1.01  1.20  1.11  0.75  0.74  1.88  0.30  1.05  1.32  1.81  1.14  5.59  3.90  1.19  1.11  2.55  0.79  1.10  1.15  1.35 | 15.15  2.40  3.31  4.67  1.25  2.11  0.89  0.52  0.45  2.65  0.46  31.84  0.28  1.89  2.00  1.97  1.70  76.21  0.61  0.22  1.05  3.80  9.49  1.69  1.03  1.62  1.72  0.51  0.67  1.77  0.63  1.01  1.36  3.13  0.89  7.80  6.70  1.24  1.81  3.43  0.66  1.76  1.55  3.11 | 1.61  0.55  0.87  0.30  0.37  1.40  0.84  1.13  1.83  0.53  2.68  0.80  0.90  0.57  0.57  0.62  0.33  0.61  0.01  BDL**  0.37  0.76  1.77  1.38  1.48  1.60  1.78  1.38  1.02  1.36  3.57  1.56  0.97  2.44  1.69  1.88  1.60  1.15  0.94  3.54  0.68  1.78  1.89  3.27 | 497.53  5.63  13.74  6.34  0.30  13.49  1.72  0.92  0.98  6.59  2.85  315.64  0.19  2.11  2.42  8.55  0.26  187.23  0.003  BDL**  0.44  9.67  155.13  39.53  1.55  3.09  3.40  0.53  0.51  4.52  0.66  1.65  1.74  13.87  1.72  81.89  41.86  1.69  1.89  30.94  0.36  3.45  3.35  13.68 | *Glycolysis*  *Inflammatory response*  *Docosahexaenoic acid metabolism*  *Arachidonic acid metabolism*  *Regulation of prostaglandin synthesis*  *Electron transport chain*  *Purine metabolism*  *Purine metabolism*  *Purine metabolism*  *ROS generation*  *FA β-oxidation*  *Glycerophospholipid metabolism*  *Cardiolipin (CL) biosynthesis*  *Aldosterone synthesis*  *Corticosterone metabolism*  *FA ω-oxidation*  *Intercellular lipid signaling*  *CL synthesis and remodeling*  *Glycerophospholipid metabolism/ intracellular*  *signal transduction*  *Cell signaling/lipid metabolism*  *Glutathione degradation*  *Aldosterone synthesis*  *Prostacyclin (PGI2) generation*  *Regulation of cellular ceramide homeostasis*  *Inflammatory response*  *Docosahexaenoic acid metabolism*  *Arachidonic acid metabolism*  *Regulation of prostaglandin synthesis*  *Regulation of prostaglandin synthesis*  *Electron transport chain*  *Purine metabolism*  *ROS generation*  *FA β-oxidation*  *Cellular signaling*  *Aldosterone synthesis*  *Prostacyclin (PGI2) generation*  *Vasodilation, antiplatelet activity*  *FA ω-oxidation*  *FA ω -oxidation*  *CL synthesis and remodeling*  *Glutathione degradation*  *DHA metab./resolution of the inflammatory*  *response back to a non-inflamed state*  *Aldosterone synthesis*  *Cellular signaling* | |

* metabolites were organized according to the type of ionization mode and pathway involved.

** below detection limit at 8 h of perfusion; † for 1^st^ clinical case; †† for 2^nd^ clinical case.

Levels of identification: **5** – annotation using xMSannotator Integrative Scoring Algorithm (details on this tool can be found in the ‘Data Handling and Statistical Analysis’ section; only features with medium-to-high confidence annotations were included); **4** – found in 2 ionization modes; **3** – putatively characterized metabolite or lipid class; **2** – MS/MS spectrum match with database (putatively annotated metabolite), **1** – chromatographic retention time (Rt), m/z, mass fragmentation pattern match with authentic standard (high-confidence metabolite identification);

**‡** Ratios of the average MS ion intensities (peak areas) between particular groups studied. All the discriminant metabolites or lipid species involved in this table met significance threshold of VIP≥1.5 and/or FDR-adjusted P<0.05.
